# Clinical characteristics and intensification patterns in subjects with “early” type 2 diabetes in Italy- an analysis from the AMD annals initiative

**DOI:** 10.1007/s00592-025-02599-9

**Published:** 2025-10-30

**Authors:** Giuseppina T. Russo, Antonio Nicolucci, Alessandro Cuttone, Antonio Ceriello, Valeria Manicardi, Alberto Rocca, Francesco Prattichizzo, Giuseppe Lucisano, Salvatore Corrao, Salvatore De Cosmo, Graziano Di Cianni, Riccardo Candido

**Affiliations:** 1 Department of Clinical and Experimental Medicine, University of Messina, Messina, Italy; 2 CORESEARCH – Center for Outcomes Research and Clinical Epidemiology, Pescara, Italy; 3 IRCCS MultiMedica, Milan, Italy; 4 AMD Foundation, Reggio Emilia, Italy; 5 G. Segalini H. Bassini Cinisello Balsamo ASST Nord, Milan, Italy; 6 Department of Health Promotion Sciences, Maternal and Infant Care, Internal Medicine and medical specialties [PROMISE], University of Palermo, Palermo, Italy; 7 Department of Medical Sciences, Scientific Institute “Casa Sollievo della Sofferenza”, San Giovanni Rotondo, Italy; 8 Diabetes Unit, Livorno Hospital, Livorno, Italy; 9 Department of Medical Surgical and Health Sciences, University of Trieste, Trieste, Italy

**Keywords:** Early, Type 2 diabetes, AMD annals, GLP-1RAs, SGLT2i

## Abstract

**Purpose:**

Characteristics defining “early” type 2 diabetes (T2D) are unclear, and type and timing of treatment intensification with glucose-lowering drugs (GLD) in these patients are understudied.

**Methods:**

Within the AMD Annals Initiative, we evaluated the prevalence and clinical characteristics of early T2D subjects, evaluated by diabetologists and defined according to a recent Delphi Consensus. Patient characteristics of early T2D subjects were compared to those of non-early patients. We also explored the time and mode of first intensification in a longitudinal cohort from 2010 to 2023.

**Results:**

Overall, 127,456 people were seen in 2023, of whom 10,700 (8.39%) showed an early phenotype. Early patients were younger, more often females, had lower HbA1c, used less cardiovascular-related drugs, and had a lower prevalence of cardiovascular disease.

**Conclusion:**

In real life, T2D could be considered as early in only ~ 8% of people. Among them, less than 10% received treatment intensification during the first year of observation, although the timing of the introduction of add-on GLD improved during time and drugs with cardiovascular benefit were often chosen as second-line GLD.

In longitudinal analysis, of 42,786 early patients initially treated with metformin, 9.37% were prescribed an add-on treatment during 12 months, more frequently represented by SGLT2i, followed by GLP1-RAs and DPP4i. Mean level of HbA1c at treatment intensification improved over time, suggesting an encouraging trend through a proactive approach.

**Supplementary Information:**

The online version contains supplementary material available at 10.1007/s00592-025-02599-9.

## Introduction

Type 2 diabetes (T2D) is one of the leading causes of morbidity and premature mortality worldwide, due to its associated long-term microvascular and macrovascular complications [1].

Hyperglycemia is strongly and independently associated with diabetes-related complications and all-cause mortality, even after adjustment for other risk factors which are frequently encountered in T2D subjects, such as hypertension and dyslipidemia [2].

In the United States there are about 1.4 million new cases of type 2 diabetes each year, while in Italy this number is equal to about 350,000 new cases per year [3, 4]. An early achiviement of proper glycemic control, i.e. an HbA1c < 7%, is associated with a decreased risk of complications [5]. Indeed, the achievement of such target has a long-term benefit on the incidence of microvascular and also cardiovascular complications over time, a phenomenon referred to as the legacy effect, which is more evident in patients with a recent diabetes diagnosis [6–9]. Accordingly, diabetes treatment guidelines recommend that the HbA1c goals should be more stringent in people with a long life expectancy, low risk of hypoglycemia, and few comorbidities [10, 11].

Despite the importance of an early targeting of T2D, there is no clear definition of the “early” diabetes phenotype. A recent consensus Delphi document for the treatment of early patients with T2D[12] defined diabetes as early when (1) the individual has a short duration of illness and potentially preserved beta cell function, (2) he/she is not on drug therapy or is on metformin alone, and (3) there is no evidence of diabetes-related damage. However, whether people selected with this approach substantially differ from the other individuals with T2D in terms of other clinical characteristics is unknown. In addition, the timing and pattern of treatment intensification for glycaemic control in this specific patient’s type is poorly studied.

Recently introduced glucose-lowering drugs, such as SGLT2i and GLP-1RAs, have a demonstrated cardiovascular and renal benefit in high-risk subjects with diabetes and in specific patients without diabetes but with cardiovascular or renal diseases [13,14]. Since recent evidence suggests that such benefit extends to people with early diabetes defined as newly diagnosed diabetes [15, 16], a prompt and widespread introduction of such drugs has been advocated [17, 18]. However, the prevalence of use of these drugs in a real-life setting of people with early T2D is unknown.

Given these premises, we explored the prevalence and the clinical characteristics of early T2D subjects, defined according to the criteria of the Delphi Consensus, in a large registry in the year 2023 [19]. In addition, we evaluated the timings and therapeutic strategies commonly adopted by diabetologists in early patients in a >10-years retrospective longitudinal analysis.

## Methods

Clinical data derive from the initiative of the Italian Association of Clinical Diabetologists [Associazione Medici Diabetologi (AMD)], established in 2004 for continuous monitoring and improvement of diabetes care [19–21]. Participating clinics use an electronic clinical record system for the everyday management of outpatients, and a software was developed to extract information from these clinical databases. Data from all participating centers are collected anonymously and centrally analyzed [19–21]. The database includes information on all patients with T2D receiving care at 296 diabetes clinics in Italy during the period between January 1 st, 2004, and December 31 st, 2023. The core data set included measurements of HbA1c, blood pressure, serum creatinine, urinary albumin excretion, total-cholesterol, low-density lipoprotein cholesterol (LDL), high-density lipoprotein cholesterol (HDL), and triglycerides. Glomerular filtration rate (eGFR) was estimated for each patient by using the Chronic Kidney Disease Epidemiology Collaboration formula derived from serum creatinine values [22]. Albuminuria was diagnosed if the urinary albumin concentration was >30 mg/L, or if the urinary albumin excretion rate was >20 µg/min, or if the urinary albumin-to-creatinine ratio was >2.5 mg/mmol in men or 3.5 mg/mmol in women. Renal risk was assessed using the KDIGO classification [23].

We conducted a cross-sectional analysis relative to the year 2023 to assess the prevalence and clinical characteristics of patients fulfilling the following definition of patients with early type 2 diabetes (T2D) [12]:

- Patients naïve to glucose-lowering therapy or treated with metformin.
- Duration of diabetes < 10 years.
- Normal albumin excretion rate.
- eGFR > 30 ml/min/1,73m^2^.
- No retinopathy.

Of note, patients were considered as “early” even in the presence of duration of T2D up to 10 years, provided that they were still treated with metformin alone, thus suggesting a slow progression of the condition and preservation of beta-cell function.

As a second step, all patients with early diabetes seen between January 1 st 2010 and December 31 st 2023 were considered, to identify those receiving treatment intensification and describe the type of treatment chosen as add-on to metformin. Furthermore, we assessed longitudinal changes in clinical parameters including HbA1c, body weight, lipid parameters, blood pressure, eGFR and albuminuria before and up to three years after the add-on. HbA1c values at the time of treatment intensification were also assessed separately in three periods: between 2010 and 2014, between 2015 and 2019, and between 2020 and 2023.

### Statistical analysis

Descriptive data are summarized as mean and standard deviation or median and interquartile range (IQR) for continuous variables and percentages for categorical variables.

Longitudinal changes in HbA1c levels are expressed as estimated mean values with their 95% confidence intervals (95%CI), derived from mixed linear models for repeated measures.

Tests were 2-sided, and a p value < 0.05 was considered statistically significant. Statistical analyses were performed with SAS software, version 9.4 (SAS Institute Inc. North Carolina, USA).

## Results

### Prevalence and clinical characteristics of “early” T2D subjects

During 2023, 127,456 patients were evaluated, for whom all the information needed to identify early patients was available. Of these, 10,700 (8.39%) were found to have early diabetes, according to the Delphi consensus definition [12]. The characteristics of patients with early and non-early diabetes are reported in Table 1. Compared to non-early patients, early patients were less often male and were younger. Moreover, about one in five cases (18.9%) had newly diagnosed diabetes (i.e. people who were referred to the diabetologist soon after the diagnosis made by the general practitioner). Subjects with early diabetes were also characterized by lower levels of HbA1c and triglycerides and slightly higher levels of total, LDL and HDL cholesterol. Only 9.3% had eGFR values below 60 ml/min, and only 1.5% were classified as being at high risk of kidney disease according to the KDIGO classification. The exclusion of such patients did not materially change the results (data not shown).

**Table 1:** Characteristics of patients with T2D in relation to the presence of early diabetes

Characteristics	Early diabetes	
	**No**	**Yes**
Number of patients	116,756	10,700
Sex (% males)	62.9	56.2
Age (years)	69.7 ± 10.4	66.5 ± 10.7
Duration of T2DM (years)	14.2 ± 9.6	3.6 ± 3.2
Newly diagnosed T2DM	4.0	18.9
Smokers	17.1	15.4
HbA1c (%)	7.1 ± 1.1	6.4 ± 0.7
Total cholesterol (mg/dl)	152.8 ± 36.5	166.0 ± 37.5
LDL cholesterol (mg/dl)	78.1 ± 30.6	90.7 ± 32.6
HDL cholesterol (mg/dl)	49.0 ± 12.8	51.7 ± 12.9
Triglycerides (mg/dl)	127.4 ± 70.6	118.0 ± 62.9
Systolic blood pressure (mmHg)	134.5 ± 18.1	133.6 ± 17.3
Diastolic blood pressure (mmHg)	76.2 ± 9.8	77.5 ± 9.7
BMI (kg/m^2^)	28.7 ± 5.4	28.3 ± 4.8
eGFR < 60 ml/min	31.8	9.3
Renal risk (KDIGO) Low risk Moderate risk High risk Very high risk	49.3 24.4 12.2 14.1	90.7 7.8 1.5 0
Lipid-lowering treatment	76.1	66.3
Anti-hypertensive treatment	72.7	59.0
Established cardiovascular disease (myocardial infarction, stroke, coronary and/or peripheral revascularization, coronary and/or peripheral bypass)	17.7	5.5
Myocardial infarction	9.3	2.6
Heart failure	4.0	0.7
TIA	1.7	0.9
Stroke	2.8	1.3

Data are expressed as mean ± standard deviation or percentages

Regarding glucose-lowering therapy, as for definition, early subjects were treated with metformin and/or diet only; in particular, 13.7% were on diet only, and 86.3% were on metformin. Early subjects were less often treated with anti-hypertensive and lipid-lowering drugs.

Despite being early, 5.5% of patients had established cardiovascular disease (CVD): 2.6% myocardial infarction, 1.3 stroke and 0.7 heart failure, although these prevalences were significantly lower when compared to subjects who did not respond to the early criteria (Table 1). Data on complications and baseline glucose lowering drugs are presented in Supplementary Table 1.

## Intensification patterns in early T2D subjects

In the period 2010–2023, 42,786 early patients initially treated with metformin alone were identified. Of these, during a median follow-up of 12 months (IQR 12–24), 4011 (9.37%) early subjects had an intensification of metformin with an add-on of at least another glucose-lowering drug.

Glucose-lowering drugs used for intensification in early patients were also investigated. Data indicates that the more frequently used classes for therapeutic intensification were SGLT2i, followed by GLP1-RAs and DPP4i. In particular, the percentage of drugs used for intensification were as follows: SGLT2i 40.4%, GLP-1RAs 30.7%, DPP4i 27.2%, insulin 5.5%, sulphonylureas 5.3%, pioglitazone 3.3%, acarbose 1.5%, glinides 0.3%.

The trend of HbA1c values ​​before and after the intensification with a second drug is reported in Table 2. On average, HbA1c values ​​at the time of the add-on were 7.28%, with a reduction of approximately 0.4% after the intensification.

**Table 2:** HbA1c changes after add-on to metformin – Estimated mean values ​​(mixed models for repeated measures)

Time	Estimated mean value (95% CI)	Mean change vs. Baseline (95% CI)	*p*-value
Baseline (before add-on)	7.28 (7.25;7.31)	Ref.	-
12 months	6.89 (6.86;6.92)	−0.39 (−0.42;−0.36)	< 0.0001
24 months	6.87 (6.83;6.91)	−0.41 (−0.46;−0.36)	< 0.0001
36 months	6.97 (6.92;7.02)	−0.31 (−0.36;−0.26)	< 0.0001

Table 3 shows the time course of clinical parameters in early subjects who had a therapeutic intensification. In addition to the reduction of HbA1c, a slight reduction in LDL cholesterol levels, systolic blood pressure, BMI and body weight was documented. A reduction in eGFR was also present, and the onset of albuminuria was registered in 13.3% of patients. Supplementary Table 2 shows temporal trends in BMI and body weight according to the drug classes used for intensification of metformin therapy. In patients receiving a GLP1-RA, average BMI decreased from 33.3 ± 5.9 kg/m^2^ at baseline to 32.4 ± 5.6 kg/m^2^ at 24 months and 32.7 ± 5.8 kg/m^2^ at 36 months. In patients receiving a SGLT2-i as intensification therapy, a slight decrease in BMI was documented, from 30.7 ± 5.5 kg/m^2^ at baseline to 30.1 ± 5.4 kg/m^2^ at 12 and 24 months and 30.2 ± 5.5 kg/m^2^ at 36 months.

**Table 3:** Add-on to Metformin in subjects with early diabetes - trends in characteristics over the years

Characteristics	Baseline	12 months	24 months	36 months
Number of patients	4,011	4,011	2,587	1,834
Total cholesterol (mg/dl)	175.6 ± 40.0	164.1 ± 37.9	162.3 ± 38.5	162.2 ± 37.5
LDL cholesterol (mg/dl)	98.7 ± 34.2	88.4 ± 32.4	86.2 ± 31.7	85.7 ± 31.6
HDL cholesterol (mg/dl)	48.5 ± 12.3	48.9 ± 12.3	49.2 ± 12.7	49.1 ± 13.0
Triglycerides (mg/dl)	148.9 ± 96.3	137.6 ± 76.0	140.1 ± 92.6	142.8 ± 84.0
Systolic blood pressure (mmHg)	134.2 ± 17.5	132.4 ± 16.7	132.2 ± 16.6	131.9 ± 16.0
Diastolic blood pressure (mmHg)	79.4 ± 9.9	78.6 ± 9.7	78.1 ± 9.3	78.2 ± 9.6
BMI (kg/m^2^)	30.9 ± 5.8	30.3 ± 5.8	30.2 ± 5.7	30.4 ± 5.7
Body weight (kg)	86.1 ± 18.6	84.6 ± 18.4	84.5 ± 18.2	85.0 ± 18.4
eGFR (ml/min)	87.4 ± 16.7	83.5 ± 21.9	82.2 ± 23.1	81.2 ± 24.1
Subjects with micro/macroalbuminuria	0	11.8	13.7	13.3

Data are expressed as mean ± standard deviation or percentages

Supplementary Table 3 reports the characteristics over time of patients with early T2D receiving metformin monotherapy and not undergoing treatment intensification. The table shows that these patients had satisfactory HbA1c levels both at baseline and throughout the observation period, thus justifying the absence of therapeutic intensification. The other parameters considered also remained stable over time, with the sole exception of a gradual decline in renal function.

**Fig. 1:**
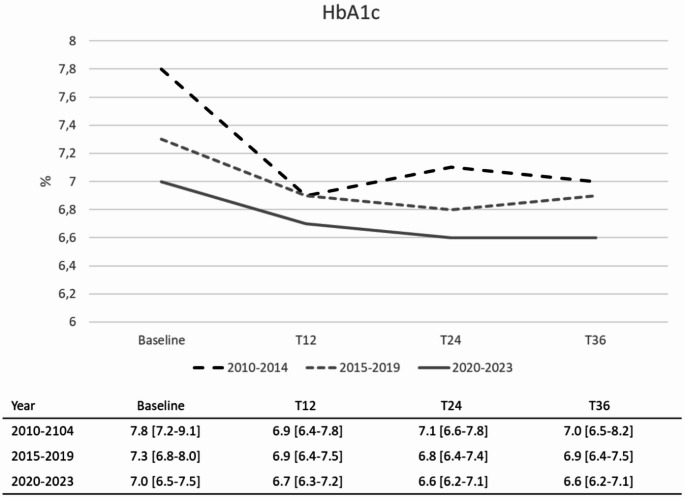
HbA1c trends before (baseline) and after add-on to metformin in three time periods

We also evaluated whether intensification strategies changed overtime in terms of HbA1c levels at the time of the first intensification. In this regard, Fig. 1 shows HbA1c values at add-on by dividing the entire observation duration into three periods. Median HbA1c values ​​at add-on decreased from 7.8% in 2010–2014 to 7.0% in the most recent period, indicating a progressive anticipation of therapeutic intensification. It should be noted that in 2010–2014 the upper quartile of HbA1c was 9.1%; in other words, a quarter of the subjects intensified treatment at HbA1c values ​​above 9.0%. In the most recent period, the value of the upper quartile decreased to 7.5%.

## Discussion

Early and intensive glycaemic control provides long-term protection against the development of vascular complications [24–26]. The importance of early treatment is supported by evidence from both clinical trials and observational studies [3, 8, 27]. 

Accordingly, both the ADA-EASD consensus and the ADA’s 2024 Standards of Medical Care [10, 11], while acknowledging the importance of the step-by-step approach, conceive the possibility of an early combination therapy for its beneficial effects on glycemic control, cardiorenal protection, and to delay treatment failure, as demonstrated by the VERIFY study [28].

Despite these recommendations, updated information on the prevalence, clinical characteristics, and therapeutic management of subjects with early T2D is sparse. Furthermore, the phenotype of early patients is poorly characterized, as is the timing of treatment intensification and prescription pattern in this population.

Knowing the prevalence and characteristics of early T2D patients could have a great relevance also in terms of health policy, in order to plan the most appropriate and timely interventions in a group of subjects in which such interventions could be disease changing.

In this respect, a recent Delphi consensus defined early T2D as people fulfilling pre-specified criteria [12]. Here we show that, applying such criteria among the 127,456 people with T2D in our register, 10,700 (8.39%) could be identified as having early diabetes. Such low prevalence might be affected by the observation that individuals with non-complicated T2D at the beginning of the disease may also be in charge of GPs^29^, while data used here derived from diabetes specialist clinics.

Of note, only one in five early patients had a newly diagnosed diabetes (18.9%), and the average duration of T2D was of 3.6 years. Also, renal risk was overall low according to the KDIGO classification, and only 9.3% had eGFR values between 60 and 30 ml/min. The exclusion of such patients did not materially change the results.

In addition, 5.5% of early patients had already established CVD, in line with existing knowledge suggesting that CVD might precede T2D or that silent diabetes could be undetected for years [29].

Among early-stage patients who were initially treated with metformin alone, approximately 10% had their therapy intensified through the addition of another agent. Mean HbA1c ​​at the time of the intensification was 7.28%, indicating that reasons behind the therapeutic change are likely go beyond the sole achievement of strict HbA1c targets. Indeed, results suggest that HbA1c values ​​at the time of intensification decreased progressively when considering three successive periods, suggesting a more proactive attitude of diabetologists and an overall amelioration of therapeutic inertia. Overall, after the intensification, we observed a reduction of approximately 0.4% of mean HbA1c. Notably, the intensification was also accompanied by a general amelioration of other CVD risk factors, including LDL cholesterol levels, systolic blood pressure, and BMI, corroborating the notion that avoiding clinical inertia is a key aspect of diabetes management [30–32]. Importantly, early patients not subjected to treatment intensification exhibited consistently good metabolic control over the study period, supporting the absence of treatment escalation.

In addition, our data suggest that the more frequently used classes for therapeutic intensification were SGLT2i, followed by GLP1-RAs and DPP4i. Indeed, SGLT2i and GLP-1Ras were prescribed in more than 40% and 30% of these patients, respectively, in line with a recent analysis on the overall 2023 AMD Annals Initiative T2D cohort [19]. This evidence suggests that, even though the beneficial effects of these GLD on people with early diabetes is only recently emerging [15, 33, 34], their use as second-line options in this population with low cardiorenal risk is already consistent. A trend toward an earlier intensification attitude in terms of HbA1c and treatment patterns similar to our results were also recently documented in a Danish nationwide study [34]. Similar data were provided also in our previous study in Italy, which reported a more common use of innovative drugs in the recent years and an overall trend in initiating a new drug at lower HbA1c levels [35]. Conversely, a recent U.S. study based on data from two large health insurance databases showed much lower prescription rates for SGLT2i and GLP-1RAs, and a higher use of sulphonylureas [36]. A possible reason explaining these results include the analyzed period (2013–2019), during which less data was available for these GLD. In addition, it might be speculated that, besides clinical considerations, other factors such as the overall organization of health systems might influence the choice of treatments of people with early T2D.

Several limitations should be considered when evaluating our results, which are innate to the observational nature of our analysis, including residual unmeasured confounders. In addition, we did not perform subgroup analyses to explore eventual heterogeneity among sexes or according to age strata or other clinical characteristics.

## Conclusions

In summary, this analysis on an updated, large cohort of people with T2D identified ~ 8% of individuals fulfilling the criteria for early diabetes. Only a minority of them received treatment intensification during the first year of observation but the timing of intervention is progressively improving in recent years. SGLT2i and GLP-1RAs were the most commonly used drugs for this purpose. These results corroborate the perceived importance of an early improvement of glycemic control in real-life and suggest the progressive diffusion of cardioprotective drugs even in subjects with T2D but without CVD nor kidney disease and/or at moderate/low risk for these complications.

## Supplementary Information

Below is the link to the electronic supplementary material.

Supplementary Material 1

Supplementary Material 2

Supplementary Material 3

## Data Availability

The datasets generated during and/or analyzed in the current study are available from the corresponding author upon reasonable request.
