# Supplementary material for: Clinical characteristics and intensification patterns in subjects with “early” type 2 diabetes in Italy- an analysis from the AMD annals initiative
Source: Acta Diabetol. 2025 Oct 30;63(3):453–9. doi: 10.1007/s00592-025-02599-9 (PMC13046648; doi:10.1007/s00592-025-02599-9)
Supplement: Supplementary file 1 — Supplementary Material 1 [file 592_2025_2599_MOESM1_ESM.pdf]

SPRINGER NATURE

Disclosure of potential conflicts of interest

Authors must disclose all relationships or interests that could have direct or potential influence or impart bias on the work. Although an author may not feel there is any conflict, disclosure of all relationships and interests provides a more complete and transparent process, leading to an accurate and objective assessment of the work. Awareness of real or perceived conflicts of interest is a perspective to which the readers are entitled. This is not meant to imply that a financial relationship with an organization that sponsored the research or compensation received for consultancy work is inappropriate. For examples of potential conflicts of interests *that are directly or indirectly related to the research* please visit:
www.springer.com/gp/authors-editors/journal-author/journal-author-helpdesk/publishing-ethics/14214

Corresponding authors of papers submitted to Acta Diabetologica
[*include name of journal*] must complete this form and disclose any real or perceived conflict of interest. The corresponding author signs on behalf of all authors.

The corresponding author will include a statement in that reflects what is recorded in the potential conflict of interest disclosure form. Please check the Instructions for Authors where to put the statement which may be different dependent on the type of peer review used for the journal.Please note that you cannot save the form once completed. Please print upon completion, sign, and scan to keep a copy for your files.
The corresponding author should be prepared to send the potential conflict of interest disclosure form if requested during peer review or after publication on behalf of all authors (if applicable).

[X] We have no potential conflict of interest.

Category of disclosure	Description of Interest/Arrangement

Article title Clinical characteristics and intensification patterns in subjects with "early" type 2 diabetes in Italy- an analysis from the AMD Annals Initiative

Manuscript No. (if you know it) ______

Corresponding author name Giuseppina T. Russo

Herewith I confirm, on behalf of all authors, that the information provided is accurate.

Author signature Giuseppe ~~Date~~ ______
