# Supplementary material for: Clinical characteristics and intensification patterns in subjects with “early” type 2 diabetes in Italy- an analysis from the AMD annals initiative
Source: Acta Diabetol. 2025 Oct 30;63(3):453–9. doi: 10.1007/s00592-025-02599-9 (PMC13046648; doi:10.1007/s00592-025-02599-9)
Supplement: Supplementary file 2 — Supplementary Material 2 [file 592_2025_2599_MOESM2_ESM.pdf]

AMD ANNALS STUDY GROUP
(in alphabetical order by Italian region and town)

| Region | Authors | Hospital | Department | Town |
|---|---|---|---|---|
| ABRUZZO | Valeria Montani, Emanuela Cannarsa, Andrea Dudiez, Martina Basilico, Vincenzo Trosini, Rosanna Pennetta | ASL di Teramo Dipartimento Discipline Mediche | UO Diabetologia | Atri (TE) |
| ABRUZZO | Deamaria Piersanti, Pietro Mercuri, Barbara Macerola, Elisabetta Di Berardino, Alessia Di Pietro, Loredana Capaldi, Lucia Di Felice | ASL 1 Abruzzo - P.O. Avezzano | UOSD Diabetologia | Avezzano (AQ) |
| ABRUZZO | Marilena Olivieri, Valter Milano, Maria Lucia Parente | Distretto Sanitario di Base di Casoli - ASL Lanciano-Vasto-Chieti | Ambulatorio Di Diabetologia | Casoli (CH) |
| ABRUZZO | Livia Santarelli, Annamaria De Mutiis, Marco Iezzi, Antonietta Sciulli, Carla Mennella, Carla Scarsellato | Presidio Ospedaliero Castel di Sangro | UOSD di Diabetologia - ASL1 Abruzzo Avezzano-Sulmona-L'Aquila | Castel di Sangro (AQ) |
| ABRUZZO | Ester Vitacolonna, Cinzia Carla Carrabs, Elena Giampietro | Ospedale Clinicizzato SS Annunziata | Diabetologia del Policlinico Chieti | Chieti (CH) |
| ABRUZZO | Maria D'Aurizio | PTA Presidio Territoriale di Assistenza Gissi | Reparto di Diabetologia | Gissi (CH) |
| ABRUZZO | Valeria Montani, Amina Di Gennaro, Angelo Cosimo Gioia, Marina Manieri, Susanna Lupidii, Sandrine Cengarle | Presidio Ospedaliero Giulianova - ASL Teramo | UO Diabetologia | Giulianova (TE) |
| ABRUZZO | Marco Giorgio Baroni, Sara Nazzarena Morgante, Antonella Zugaro | Ospedale San Salvatore - L'Aquila ASL 1 Abruzzo | Diabetologia e Malattie Metaboliche | L'Aquila (AQ) |
| ABRUZZO | Fiore Pelliccione, Alessio Martorella, Tiziana Rapino | Ospedale "F. Renzetti", ASL 02 Abruzzo | Servizio di Diabetologia | Lanciano (CH) |
| ABRUZZO | Agostino Consoli, Gloria Formoso, Merilds Taraborrelli, Marica Milo, Giulia Di Dalmazi, Sara Coluzzi, Fabrizio Febo | AUSL Pescara Ospedale Civile Santo Spirito | UOC Territoriale Endocrinologia e Metabolismo | Pescara (PE) |
| ABRUZZO | Benedetta Martin | ASL Pescara Presidio Ospedaliero di Popoli | Ambulatorio Diabetologia UOC Medicina Interna | Popoli (PE) |
| ABRUZZO | Rossella Romano, Patrizia Di Fulvio, Antonio Macrillante, Maria Laura Daniele, Sonia Cicconi, Caterina Raimondo | ASL 4 Teramo Ospedale Val Vibrata | UOS Diabetologia | Sant' Omero (TE) |
| ABRUZZO | Livia Santarelli, Annamaria De Mutiis, Marco Iezzi, Giovanni Cavaliere, Luigina Ventresca, Debora Gambina, Luigina Spadorcia, Patrizia Di Giovine | Ospedale Civile SS Annunziata - ASL1 Abruzzo Avezzano-Sulmona-L'Aquila | UO di Diabetologia - Sulmona | Sulmona (AQ) |

| Region | Authors | Hospital | Department | Town |
|---|---|---|---|---|
| ABRUZZO | Paola Romagni, Terrenzio Marco, Antonella De Gregorio, Guido Malatesta, Roberto Di Stefano, Gabriella Quaranta | Presidio Ospedaliero di Teramo | UOS Diabetologia | Teramo (TE) |
| ABRUZZO | Mariarosaria Squadrone, Grazia Giovanna La Verghetta, Maria D'Aurizio, Donatella Luciani, Concetta Massa, Giovina Lapergola, Carmelina Pannunzio, Sabrina Sciascia, Nadia D'Ottavio | Nucleo Operativo Distrettuale Vasto | Servizio Diabetologico C/0 Nod Vasto 1 piano | Vasto (CH) |
| CALABRIA | Rosanna Piro | Ospedale San Francesco di Paola | Reparto di Diabetologia | Paola (CS) |
| CALABRIA | Luigi Puccio, Paola Sarnelli, Antonietta Comito | Azienda Ospedaliera Pugliese-Ciaccio | SOC Diabetologia - Endocrinologia | Catanzaro (CZ) |
| CALABRIA | Eugenio Alessi, Concetta Nadia Arico', Claudia Ferraro, Maria Angela Sculli | A.O. Bianchi Melacrino Morelli Presidio Ospedaliero Morelli | UOC Diabetologia ed Endocrinologia | Reggio Calabria (RC) |
| CALABRIA | Celestino Giovannini, Daniela Cristiano | ASP 5 Reggio Calabria Polo Sanitario Nord | Servizio di Diabetologia | Reggio Calabria (RC) |
| CALABRIA | Alessandra Paris, Giovanni Perrone | Polo Sanitario RC-Sud ASP 5 | Servizio Territoriale di Diabetologia | Reggio Calabria (RC) |
| CALABRIA | Giuseppe Armentano, Mariagrazia Restuccia, Anton Giulio Ametrano, Mariangela Rubino, Alfonso Mele, Arcangelo Maurizio Palermo, Anita Armentano, Mariarosaria Ciccopiedi Ciccopiedi, Teresa Adimari, Davide Antonio Aquino, Antonella Arnone, Maria Elvira Tavernise | Centro Diabetologico DEA - ASP Cosenza | Accreditato SSN - ASP Cosenza | Rossano Calabro (CS) |
| CAMPANIA | Andrea Del Buono, Maria Barone, Patrizia Guacci, Nicola Ferraro, Luigina Imbrogno, Valeria Aliperta, Lucia Marciano | ASL Caserta - Distretto 12 | Centro Diabetologico | Caserta (CE) |
| CAMPANIA | Mario Tarquini, Rosa Di Fraia | ASL Caserta - Distretto 14 | Centro Diabetologico | Cellole (CE) |
| CAMPANIA | Mario Laudato, Maria Carmen Passariello | ASL Caserta - Distretto 13 | Ambulatorio di Diabetologia | Maddaloni (CE) |
| CAMPANIA | Claudio Lambiase, Teresa Di Vece, Massimo Fezza, Carmela Giordano, Flora Leo, Iolanda Marchese, Luigi Gargiulo, Linda D'Amato, Vincenzo Pagliara, Luigi Sardelli, Gabriella Nosso, Maurizio Mauriello | Ospedale Curteri G. Amico DS 67 - ASL SA | Centro Diabetologico | Mercato S. Severino (SA) |
| CAMPANIA | Stefano De Riu | ASL NA 1 - Distretto sanitario 33 | Servizio Diabetologico | Napoli (NA) |

| Region | Authors | Hospital | Department | Town |
|---|---|---|---|---|
| CAMPANIA | Emilia Martedi' | Centro Diagnostico San Ciro | Centro AID | Portici (NA) |
| CAMPANIA | Gelsomina Capuano, Alessandra Cantillo, Lea Lo Conte, Giovanni Attanasio, Mariaconsiglia Landi | ASL Salerno Centro Diabetologico Distretto 66 Ospedale Vernieri | Centro Diabetologico | Salerno (SA) |
| CAMPANIA | Francesca Innelli, Angela Cassese, Valentino De Stefano, Piera Grammaldo, Luigi Scevola, Emanuela Petraglia, Angelo Vistocco | CAD 70 II Livello DS Vallo della Lucania/Agropoli ASL SALERNO | Ambulatorio Diabetologia | Vallo della Lucania (SA) |
| EMILIA ROMAGNA | Biagio Oliviero, Marcello Monesi | Poliambulatorio di Portomaggiore | UOC Diabetologia Territoriale Ferrara | Argenta - Portomaggiore (FE) |
| EMILIA ROMAGNA | Michele Salvatore Grimaldi, Valentina Lo Preiato, Simona Moscatiello, Danilo Ribichini, Sara Flamigni, Uberto Pagotto, Guido Di Dalmazi | AOU di Bologna - IRCCS Policlinico di Sant'Orsola | Endocrinologia e prevenzione e cura del diabete | Bologna (BO) |
| EMILIA ROMAGNA | Giorgia Prampolini, Marzia Turilli, Licia Notari, Ugo Aldo Pagliani | Ospedale di Castelnovo ne' Monti | SOS Diabetologia | Castelnovo ne' Monti (RE) |
| EMILIA ROMAGNA | Vincenzo Maria Monda, Marcello Monesi | Poliambulatorio di Cento | UOC Diabetologia Territoriale Ferrara | Cento (FE) |
| EMILIA ROMAGNA | Elisabetta Bergami, Marcello Monesi | Ospedale di Codigoro | UOC Diabetologia Territoriale Ferrara | Codigoro - Comacchio (FE) |
| EMILIA ROMAGNA | Elisa Usberti | AUSL Parma Ambulatorio Casa della Salute Collecchio | Ambulatori Diabetologici Distretto Sud - Est | Collecchio (PR) |
| EMILIA ROMAGNA | Carlo Percudani, Angela Muoio, Gisella Boselli, Nicoletta Orlandi, Antonella Guberti | AUSL PARMA Polo Sanitario di Colorno | Ambulatorio Diabetologico | Colorno (PR) |
| EMILIA ROMAGNA | Anna Rita Carli, Dinatolo Elisa, Marcello Monesi | Poliambulatorio di Copparo | UOC Diabetologia Territoriale Ferrara | Copparo (FE) |
| EMILIA ROMAGNA | Bruna Milli, Petros Tsamatropoulos, Giulia Bellei, Daniela Di Renzo | Ospedale di Correggio | SOS Diabetologia | Correggio (RE) |
| EMILIA ROMAGNA | Maria Simona Termine, Elena Casadio, Paolo Di Bartolo | PO di Faenza - AUSL della Romagna | UO Diabetologia Ravenna/Faenza, Dip. Internistico | Faenza (RA) |
| EMILIA ROMAGNA | Marcello Monesi, Anna Rita Carli, Francesca Lugli, Mariaenrica Bellio, Serena Galvan, Dinatolo Elisa | AUSL Ferrara | UOC Diabetologia Territoriale Ferrara | Ferrara (FE) |
| EMILIA ROMAGNA | Antonella Guberti, Gisella Boselli, Nicoletta Orlandi, Elisa Gatti, Cristina Amadè | Ospedale di Fidenza | UO Medicina Interna | Fidenza (PR) |

| Region | Authors | Hospital | Department | Town |
|---|---|---|---|---|
| EMILIA ROMAGNA | Bruna Milli, Silvia Pilla, Giulia Bellei, Daniela Di Renzo, Petros Tsamatropoulos | Ospedale di Guastalla | SOS Diabetologia | Guastalla (RE) |
| EMILIA ROMAGNA | Anna Vacirca, Rita Manini | Ospedale Civile Nuovo di Imola | Dipartimento Medico Oncologico | Imola (BO) |
| EMILIA ROMAGNA | Diletta Ugolotti, Elisa Usberti, Sonia Erta, Tiziana Cadossi, Daniela Pedrini | Ausl Parma Ambulatorio Casa della Salute di Langhirano | Ambulatorio Diabetologia | Langhirano (PR) |
| EMILIA ROMAGNA | Alessandra Luberto, Paolo Di Bartolo | PO di Lugo - AUSL della Romagna | UO Diabetologia Ravenna/Lugo, Dip. Internistico | Lugo (RA) |
| EMILIA ROMAGNA | Massimo Michelini, Rosa Maria Trianni, Francesca Borghi, Elena Capuano, Marika Iemmi, Serena Davoli, Paola Pedrini | Ospedale di Montecchio | SOS Diabetologia | Montecchio (RE) |
| EMILIA ROMAGNA | Riccardo Bonadonna, Monica Antonini, Angela Vazzana, Valentina Moretti, Raffaella Aldigeri, Alessandra Dei Cas | AOUPR Azienda Ospedaliera Universitaria Parma - Dip. di Medicina generale e specialistica | UOC di Endocrinologia e Malattie del metabolismo | Parma (PR) |
| EMILIA ROMAGNA | Antonella Guberti, Gisella Boselli, Nicoletta Orlandi, Clelia Di Secli, Diletta Ugolotti, Angela Muoio, Daina Filippi, Eleonora Zambelli, Celeste Di Santi | AUSL Parma Polo Sanitario Pintor Molinetto Parma | Ambulatorio Diabetologico | Parma (PR) |
| EMILIA ROMAGNA | Silvia Haddoub, Maria Grazia Magotti, Francesco Maccanelli, Federico Cioni, Michele Riva, Maddalena Micheli | Azienda Ospedaliero-Universitaria di Parma | Reparto di Trattamento intensivo Del Diabete e delle sue Complicanze | Parma (PR) |
| EMILIA ROMAGNA | Paolo Di Bartolo, Francesca Pellicano, Arianna Mazzotti, Cipriana Sardu, Maria Turchese Caletti, Chiara Caselli | AUSL della Romagna | Reparto di Diabetologia | Ravenna (RA) |
| EMILIA ROMAGNA | Elisa Manicardi, Lisa Bonilauri, Eliana Gardini, Miriam Parisi, Alessandro Lo Ioco, Prisco Sbordone, Rosa Trianni, Antonella Paola Sacco, Eles Notari, Rita Montedoro, Sara Pingani, Roberta Prandi, Sussanna Gelosini, Laura Lombardi, Maria Cardaci, Maria Gloria Mercati, Federica Ferrari, Francisca De Barros | Casa della Salute, Distretto di Reggio Emilia | SOS Diabetologia | Reggio Emilia (RE) |
| EMILIA ROMAGNA | Ugo Aldo Pagliani, Monica Camporesi, Antonella Rabitti, Anna Maria Ferrarioli, Simona Bodecchi, Susanna Valenti | AUSL di Reggio Emilia-Ospedale di Scandiano | SOS Diabetologia | Scandiano (RE) |
| EMILIA ROMAGNA | Elisa Usberti, Daniela Pedrini | AUSL Parma Ambulatorio casa della Salute di Traversetolo | Ambulatorio di Diabetologia | Traversetolo (PR) |

| Region | Authors | Hospital | Department | Town |
|---|---|---|---|---|
| FRIULI VENEZIA GIULIA | Mario Velussi | Casa di Cura Pineta del Carso | Ambulatorio di Diabetologia | Aurisina (TS) |
| FRIULI VENEZIA GIULIA | Barbara Catone, Livia Cargnelutti, Francesca De Filippi, Emanuela Tavagnacco | Ospedale Civile San Michele | Medicina Generale - Ambulatorio Diabetologico | Gemona del Friuli (UD) |
| FRIULI VENEZIA GIULIA | Daria Albini, Fabrizio Santoro, Giuseppe Felace, Lorj Mongiat, Nicoletta Petracco, Maria Elena Salvador, Elisa Norio, Serena Battistutti, Manola Nicoletti | AAS5 Friuli Occidentale Presidio Ospedaliero Santa Maria degli Angeli sede di Maniago | Diabetologia | Maniago (PN) |
| FRIULI VENEZIA GIULIA | Paolo Bordin, Luciana Dotto, Maurizio Sancandi, Viviana Casarsa, Elena Rosso, Roberto Da Ros | AAS n.3 Alto Friuli - Collinare-Medio Friuli O.C. Sant' Antonio | Ambulatorio di Diabetologia - UO Medicina | S. Daniele del Friuli (UD) |
| FRIULI VENEZIA GIULIA | Cesare Miranda, Giorgio Zanette, Elena Rinaldo | Ospedale Civile Di Sacile AAS5 Pordenone | Reparto di Diabetologia | Sacile (PN) |
| FRIULI VENEZIA GIULIA | Manola Nicoletti, Martina Grando, Rita Centis, Paola Sette, Roberta Basile, Renata Rainis | Azienda Ospedaliera Universitaria S. Maria della Misericordia sede di San Vito al Tagliamento | Ambulatorio di Diabetologia | San Vito al Tagliamento (PN) |
| FRIULI VENEZIA GIULIA | Giuseppe Felace, Daria Albini, Fabrizio Santoro, Agnese Fasano, Ilenia Donaduzzi | Presidio Ospedaliero S. Giovanni dei Battuti | Ambulatorio di Diabetologia | Spilimbergo (PN) |
| FRIULI VENEZIA GIULIA | Riccardo Candido, Chiara Gottardi, Alessandra Petrucco, Elena Manca, Iris Buda, Michela Casson, Elisabetta Tommasi, Katja Tercelj, Augusta Toso, Silvana Cum, Elisa Del Forno, Elena Mellini, Veronica Fragiacomo, Cinzia Sain, Alessandra Bresciani, Anna Maria Valiani, Letizia Dezzoni, Annamaria Ciuk, Fabrizia Banello | Azienda Sanitaria Universitaria Integrata di Trieste | SS Centro Diabetologico Distretto 3 | Trieste (TS) |
| FRIULI VENEZIA GIULIA | Silvia Galasso, Sandra Agus, Maria Carpentieri, Silvia Maria Sciannimanico, Veronica Tonelli, Cristina Sartori, Francesca Vidotti, Giovanna Cassan, Annarita Paccini, Graziana Fabbro, Marina Armellini, Angela Rutigliano, Rita Brovedani, Raffaella Feresin, Romina Nadalin, Monica Loszach, Claudia Cossa | Azienda Sanitaria Universitaria Friuli Centrale (ASUFC) | SOC di Endocrinologia | Udine (UD) |
| LAZIO | Paolo Fiorentini, Claudio Grande, Chiara Ottaviani | ASL Viterbo - Ospedale di Acquapendente | Ambulatorio di diabetologia | Acquapendente (VT) |
| LAZIO | Grazia Pia Ricciardi | ASL Latina Distretto 1 Aprilia | Servizio diabetologia | Aprilia (LT) |
| LAZIO | Maria Cristina Ribaudo, Laura Proietti Pannunzi, Rodolfo Tramonta, Fabio | ASL RM 4 | Diabetologia | Capena (RM) |

| Region | Authors | Hospital | Department | Town |
|---|---|---|---|---|
| | Mangalaviti, Lara Di Sante, Elisabetta Meloni, Stefania Ciriello, Elisabeth Ruth Serralazo, Anna Lorenzetti, Valentina Crocetti, Elizabeth Ruth Serralazo | | | |
| LAZIO | Graziano Santantonio, Lucrezia Russo, Olimpia Bitterman, Chiara Moretti, Alessandra Zappaterreno, Sabrina Agostini, Lina Lottatori, Elisa Costanzo, Debora Fabbretti, Valeria De Persio, Valentina Di Fiordo | Presidio Ospedaliero San Paolo - Civitavecchia | UOSD Diabetologia | Civitavecchia (RM) |
| LAZIO | Roberta Gaudioso, Vittoria Vaccari | ASL Roma 5 - Ospedale di Colleferro | UOC Medicina Interna e Geriatria | Colleferro (RM) |
| LAZIO | Francesco De Meo, Daniela Fiore, Claudia Iannone, Monika Latella, Franca Mastrobattista, Generosa Pannozzo | Ospedale di Fondi | Reparto di Diabetologia | Fondi (LT) |
| LAZIO | Francesco De Meo, Rossella Fabiano, Nunzia Brusca, Claudio Caiazzo, Vincenza Mariateresa Amalia Molinari, Marina Mercuri, Maria Pia Battista, Sara Migliozzi | Presidio Ospedaliero di Gaeta | SC Diabetologia ed Endocrinologia | Gaeta (LT) |
| LAZIO | Raffaele Scalpone, Sandro Lo Pinto, Glauco Messina | INI Istituto Neurotraumatologico Italiano | Ambulatorio di Diabetologia/Reparto di Medicina Generale | Grottaferrata (RM) |
| LAZIO | Lorena Mancini, Giulia Bassotti, Caterina Saponara, Olimpia Bitterman, Giuseppe Pasimeni, Gloria Cornacchiola, Debora Di Biagio, Rita Donnino, Celeste Vallesi, Fabrizio Farris, Fabio Mangalaviti | ASL Roma 4 - Casa della Salute Ladispoli/Cerveteri | Diabetologia | Ladispoli (RM) |
| LAZIO | Alessandra Di Flaviani, Angela Carlone, Lucia Fontana, Claudio Caccamo, Alessia Ventricini | ACISMOM | Centro Diabetologico Latina | Latina (RM) |
| LAZIO | Gaetano Leto, Danila Capoccia, Gloria Guarisco, Frida Leonetti | Ospedale Santa Maria Goretti | UOC di Diabetologia Universitaria | Latina (RM) |
| LAZIO | Anna Rita Aleandri, Silvia Caprioli, Giuseppina Beretta Anguissola, Fabrizia Toscanella, Maria Virginia Guidi, Maria Grazia Pipitone, Eleonora Pomponi, Maria Rosaria Faraglia, Verena Lilli, Cristina Giagnoli, Martina Foffo | Ospedale San Camillio De Lellis -ASL Rieti | Medicina 2 - Diabetologia | Rieti (RI) |
| LAZIO | Maria Letizia Bruschi, Roberta Parco | PDTA Diabete | Percorso Diabetologico | Roma (RM) |
| LAZIO | Raffaella Buzzetti, Ernesto Maddaloni, Luca D'Onofrio, Lucia Coraggio, Carmen Mignona, Renata Risi, Angela Balena, Silvia Pieralice | Azienda Ospedaliera Universitaria Policlinico Umberto I | UOD di Diabetologia | Roma (RM) |

| Region | Authors | Hospital | Department | Town |
|---|---|---|---|---|
| LAZIO | Paola D'Angelo, Silvia Carletti, Angela Del Prete, Santina Abbruzzese, Maria Altomare, Teresa Mondello, Veronica Tamburri, Roberta Lancione, Marianna Battimelli, Daniela Finora, Salvatore Valentino, Severino Malizia, Gabriella Ceccarelli | Ospedale Sandro Pertini | UO Diabetologia | Roma (RM) |
| LAZIO | Alessandra Di Flaviani, Stefania Angotti, Barbara Carabba, Carmen Mignogna, Sara Sterpetti, Francesco Saverio Floridi, Maddalena Ragazzo | ACISMOM Palmiro Togliatti | Centro Diabetologico Palmiro Togliatti | Roma (RM) |
| LAZIO | Alessandra Di Flaviani, Valentina Izzo, Rossella Fabiano, Alessio Maiorino, Stefano Colangelo, Roberto Gagliardi, Fabio Colletti | ACISMOM | Centro Diabetologico Concordia | Roma (RM) |
| LAZIO | Alessandra Di Flaviani, Diana Corradini, Lucilla Gagliardi, Maria Cristina Gentile, Maria Neve Hirsch, Silvia Pieralice, Francesca Piccirilli, Roberta Pisano | ACISMOM | Centro Diabetologico Camillo Negro | Roma (RM) |
| LAZIO | Danila Fava, Maria Cassone Faldetta, Fulvia De Luca, Giuliana Leacche, Anna Conidi, Rossella Guarino, Vanessa Maini, Valentina Perfili, Cristina Sacchini, Anna Tesei | A.O. S. Giovanni Addolorata Presidio Ospedaliero Santa Maria | UOSD Endocrinologia e Diabetologia | Roma (RM) |
| LAZIO | Adriana Avolio, Michela Brunetti, Gea Ciccarelli, Francesca Cinti, Gianfranco Di Giuseppe, Shawn Gugliandolo, Teresa Mezza, Simona Moffa, Cassandra Morciano, Serena Rotunno, Laura Soldovieri, Amelia Splendore, Valentina Fragale, Ilaria Improta, Andrea Giaccari | Fondazione Policlinico Agostino Gemelli IRCCS | Centro per le malattie endocrine e metaboliche | Roma (RM) |
| LAZIO | Susanna Morano, Tiziana Filardi, Vittorio Venditti, Enrico Bleve, Antonella Valente, Caterina Saponara | Policlinico Umberto I, Sapienza Università di Roma | Dipartimento di Medicina Sperimentale UOS Complicanze del diabete | Roma (RM) |
| LAZIO | Lelio Morviducci, Daniela Cappelloni, Lina Lardieri, Anna Ciarmatori, Claudia Brufani, Ilaria Giordani, Tiziana Santucci, Gabriella Del Monte, Franca Rauseo, Patrizia Alini, Daniela Chiodi, Catia Pietrangeli, Maria Elena Antonazzi, Romina Di Mauro, Carolina Francesca Bolli | Azienda Ospedaliera San Filippo Neri | UOD Diabetologia | Roma (RM) |
| LAZIO | Lelio Morviducci, Natalia Visalli, Mauro Rossini, Maria Giuseppina Migneco, Ida La Cesa, Patrizia Cortesi, Orietta Pannozzo, Umberto Gazzarini, Romina Ralli, Claudia Leonoro, Claudia Righini, Bruna Facchini, Francesca Capata, Silvia Testa | Ospedale Santo Spirito | UOC Diabetologia | Roma (RM) |
| LAZIO | Lelio Morviducci, Fabiana Lanti, Sabrina Spera, Enrica Salomone, Cinzia Riccobono, Marco Pietrantoni, Katia Minchella, Anna D'Ubaldi, Giulia Bellini | Ospedale Nuovo Regina Margherita | UOC Diabetologia | Roma (RM) |

| Region | Authors | Hospital | Department | Town |
|---|---|---|---|---|
| LAZIO | Fabiana Picconi, Marika Menduni, Benedetta Russo, Daniela Ceccaroni, Patrizia Borboni, Giuseppe Vanceri, Valentina Tommasi, Anna Maria Orlando, Elisabetta Fiorani, Simona Frontoni | Ospedale Isola Tiberina - Gemelli Isola | UOS Endocrinologia | Roma (RM) |
| LAZIO | Francesco Sabetta, Concetta Suraci, Laura Borgognoni, Antonella Caroli, Francesca Silvestri, Simona Ballacci, Letizia Mariani, Patrizia Galante | Eurosanita S.p.A. - Policlinico Casilino | Ambulatorio di Diabetologia - UO Medicina Interna | Roma (RM) |
| LAZIO | Claudio Tubili, Angelo Lauria Pantano, Maria Rosaria Nardone | AO San Camillo Forlanini | UOSD Diabetologia | Roma (RM) |
| LAZIO | Pasquale Di Perna, Patrizia Sperti, Chiara Pecchioli, Laura Giurato, Massimiliano Caprio, Simona Zaccaria, Sium Wolde Sellasie, Isabella Nardone, Luigi Uccioli | Ospedale CTO | UOC Endocrinologia e Malattie Metaboliche | Roma (RM) |
| LAZIO | Claudio Ventura, Vittoria Bonato, Marzia Bongiovanni, Esmeralda Borrello, Emma Condorelli, Angela Napoli, Sabrina Coen, Sabrina Braucci | Ospedale Israelitico | UOS Endocrinologia e Malattie Metaboliche | Roma (RM) |
| LAZIO | Anita De Ciocchis, Francesco Malci, Paolo Martini, Manuela Fiducia, Chiara Moscatelli, Emanuela Storace | ASL RM 5 Presidio Ospedaliero A. Angelucci | Medicina Interna | Subiaco (RM) |
| LAZIO | Paolo Fiorentini, Chiara Ottaviani, Teresa Puggioni, Rossana Bernabei, Lita Brunori, Monica Napoli, Valentina Perciballi | ASL Viterbo - Ospedale di Tarquinia | Ambulatorio di diabetologia | Tarquinia (VT) |
| LAZIO | Francesco De Meo, Daniela Fiore, Claudia Iannone, Anna Pacilio, Catia Palmacci, Amelia Ersilia Traversa | Ospedale di Terracina Latina | Reparto di Diabetologia | Terracina (LT) |
| LAZIO | Vincenzo Fiore, Alessandra Barucca, Giovanni Carbotta, Sonia Barraco, Antonio Angelucci, Antonella Di Pasquali, Maurizio Palmieri, Antonella Poggi, Cinzia Sforza, Anna De Paolis, Dionisia Carinella, Paola Botta, Giuseppina Runieri, Alessandra Cianfoni | ASL Roma 5 | UOSD Diabetologia - Endocrinologia | Tivoli (RM) |
| LAZIO | Alessandra Di Flaviani, Michela Dainelli, Lucia Fontana | ACISMOM Centro Diabetologico Di Viterbo | Centro Diabetologico di Viterbo | Viterbo (VT) |
| LAZIO | Paolo Fiorentini, Claudia Arnaldi, Alfonsina Chiefari, Tittania Musella, Chiara Ottaviani, Cinzia Fiorillo, Anna Montebove, Simona Vincenti, Davide Tosini, Susanna Venanzi | ASL Viterbo | Centro Diabetologico Aziendale | Viterbo (VT) |
| LIGURIA | Valeria Ghigliotti | Ospedale La Colletta | SC Diabetologia e Malattie Metaboliche ASL3 GENOVESE | Arenzano (GE) |

| Region | Authors | Hospital | Department | Town |
|---|---|---|---|---|
| LIGURIA | Paola Ponzani, Maria Rosaria Falivene, Danilo Conti, Valentina Bullara | ASL 4 Liguria | SSD Diabetologia e Malattie Metaboliche | Chiavari (GE) |
| LIGURIA | Francesca Annunziata, Elena Nazzari, Enrico Torre | Poliambulatorio Martinez di Pegli | SC Diabetologia e Malattie Metaboliche ASL3 GENOVESE | Genova (GE) |
| LIGURIA | Micaela Battistini, Arianna Cesarone, Eleonora Monti, Maurizio Patrome, Alberto Rebora, Ernesto Abramo | CDC Fiumara | SC Diabetologia e Malattie Metaboliche ASL3 GENOVESE | Genova (GE) |
| LIGURIA | Eleonora Ambrosetti, Francesca Cecoli, Francesca Fabbri, Valeria Albanese, Paola Angela Cichero | CDC Quarto Via Maggio | SC Diabetologia e Malattie Metaboliche ASL3 GENOVESE | Genova (GE) |
| LIGURIA | Laura Veronica Camerieri, Roberta Guido | Poliambulatorio Largo 12 Ottobre | SC Diabetologia e Malattie Metaboliche ASL3 GENOVESE | Genova (GE) |
| LIGURIA | Giovanni Careddu, Miryam Talco | Palazzo della salute Sant'Antonio di Recco | SC Diabetologia e Malattie Metaboliche ASL3 GENOVESE | Recco (GE) |
| LIGURIA | Luca Lione, Mario Monachesi, Amelia Casalini, Simona Oddera, Monica Cirone | ASL 2 Savonese | Ambulatorio di Diabetologia Territoriale | Savona (SV) |
| LIGURIA | Luca Lione, Gigi Bocchio, Marta Colombino, Giancarlo Markic, Monica Cirone | UCP Savona Ponente | Ambulatorio di Diabetologia | Savona (SV) |
| LOMBARDIA | Stefano Fazion, Maria Luisa Spina, Antonio Maria Labate, Anna Melegari, Maria Elena Cerutti | ASST-Mantova, Ambulatorio di Asola | SSD di Diabetologia e Malattie Metaboliche | Asola (MN) |
| LOMBARDIA | Stefano Fazion, Alessandra Malaspina, Virna Pasi, Monica Rizzardi, Cristina Grandi, Cristiana Filippini | ASST-Mantova, Ambulatorio di Bozzolo | SSD di Diabetologia e Malattie Metaboliche | Bozzolo (MN) |
| LOMBARDIA | Elena Cimino, Barbara Agosti, Sara Madaschi, Cristina Mascadri, Giulia Massari, Bernadetta Pasquino, Eugenia Resmini, Emanuela Zarra, Angela Girelli | ASST -Spedali Civili di Brescia | UOC Medicina Generale ad indirizzo Diabetologico | Brescia (BS) |
| LOMBARDIA | Nicoletta Clotilde Dozio, Ida Mangone | ASST della Brianza | SSD di Malattie Endocrine, del Ricambio e della Nutrizione | Carate (MB) |
| LOMBARDIA | Valeria Guazzoni, Gloria Groppelli, Andrea Carbone, Martina Molteni, Linda Minelli, Sara Lodigiani, Simona Devecchi, Cristina Assandri, Emilia Oleari, Silvia Breviglieri, Flavia Sormani, Rosalba Muleddu | ASST di Lodi | Ambulatorio di Casalpusterlengo | Casalpusterlengo (LO) |

| Region | Authors | Hospital | Department | Town |
|---|---|---|---|---|
| LOMBARDIA | Stefano Fazion, Maria Luisa Spina, Anna Melegari, Maria Elena Cerutti | ASST-Mantova, Ambulatorio di Castel Goffredo | SSD di Diabetologia e Malattie Metaboliche | Castel Goffredo (MN) |
| LOMBARDIA | Alberto Rocca, Paola Galli, Davide Attilio Ghelfi, Giovanna Squiccimarro, Andrea Magistro, Maria Iagulli, Monica Castellan, Rosaria Suriano, Laura Pessina, Sara Fabbri, Chiara Lessa, Sabrina Chirone, Giuseppina De Felice | Ospedale E. Bassini ASST Nord Milano | SS di Diabetologia e Malattie Metaboliche | Cinisello Balsamo (MI) |
| LOMBARDIA | Valeria Guazzoni, Gloria Groppelli, Andrea Carbone, Linda Minelli, Martina Molteni, Sara Lodigiani, Simona Devecchi, Cristina Assandri, Emilia Oleari, Silvia Breviglieri, Flavia Sormani, Rosalba Muleddu | ASST di Lodi Ospedale di Codogno | Ambulatorio di Diabetologia e Medicina | Codogno (LO) |
| LOMBARDIA | Cinzia Massafra, Maddalena Torresan, Grazia Ferro | ASST Nord Milano | Presidio dei Poliambulatori - Ambulatorio di Cologno Monzese | Cologno Monzese (MI) |
| LOMBARDIA | Angelo Alessandro Beretta, Luigi Liparulo, Nicola Lanzo | Ospedale Classificato Valduce | UO Diabetologia ed Endocrinologia | Como (CO) |
| LOMBARDIA | Chiara Mauri, Anna Bogani, Francesca Pesenti, Clemente Giglio, Paolo Elli, Carrano Giuseppe, Mariagrazia Tettamanzi, Pozzoli Raffaella, Lorena Airaghi, Elena Zardi | ASST Lariana - Azienda Ospedaliera Sant'Anna | Diabetologia | Como (CO) |
| LOMBARDIA | Valeria Valdes | ASST RHODENSE | Ambulatorio di Diabetologia - Poliambulatorio Corsico | Corsico (MI) |
| LOMBARDIA | Sergio Di Lembo, Rosa Moretta, Patrizia Ruggeri | ASST Cremona - Presidio Ospedaliero di Cremona | CAD Centro Diabetologico | Cremona (CR) |
| LOMBARDIA | Paolo Marenco, Rosana Gaiofatto, Maria Albanese | ASST Nord Milano | Presidio dei Poliambulatori - Ambulatorio di Cusano Milanino | Cusano Milanino (MI) |
| LOMBARDIA | Ida Mangone, Emanuele Spreafico, Annalisa Giancaterini, Agnese Mottadelli, Anna Cattaneo | ASST della Brianza | SSD di Malattie Endocrine, del Ricambio e della Nutrizione | Desio (MB) |
| LOMBARDIA | Laura Molteni, Giuseppe Marelli, Luciano Brambilla, Catiuscia Magni, Cornelia Iamandei | Ospedale Sacra Famiglia - Fatebenefratelli Erba | Centro Ambulatoriale Diabetologia Endocrinologia Cura delle Malattie Metaboliche | Erba (CO) |
| LOMBARDIA | Giuseppina Zaltieri, Donata Richini, Michele Squassina, Roberto Strazzeri | ASST Valcamonica | UOSS Diabetologia e Malattie del Metabolisimo | Esine (BS) |

| Region | Authors | Hospital | Department | Town |
|---|---|---|---|---|
| LOMBARDIA | Pierangelo Baro, Ida Mangone, Nicoletta Clotilde Dozio | ASST della Brianza | SSD di Malattie Endocrine, del Ricambio e della Nutrizione | Giussano (MB) |
| LOMBARDIA | Valeria Guazzoni, Gloria Groppelli, Andrea Carbone, Martina Molteni, Linda Minelli, Sara Lodigiani, Simona Devecchi, Silvia Breviglieri, Emilia Oleari, Cristina Assandri, Flavia Sormani, Rosalba Muleddu | ASST di Lodi Ospedale Maggiore di Lodi | Diabetologia e Endocrinologia | Lodi (LO) |
| LOMBARDIA | Stefano Fazion, Francesca Saggiani, Alessandra Malaspina, Maura Bosi, Alessia Sanguanini, Roberta Nuvolari, Simonetta Chiarucci, Raffaella Antoniazzi, Paola Cristanini, Elena Pierobon, Laura Paltrinieri, Roberta Casella, Simonetta Moscatelli, Rita Nardi, Cristina Pavan | ASST-Mantova, Ospedale Carlo Poma | SSD di Diabetologia e Malattie Metaboliche | Mantova (MN) |
| LOMBARDIA | Luigi Sciangula, Alessandra Ciucci, Baldassare Grassa, Giacomo Sturniolo, Emanuela Simona Olivo, Maria Grazia Magni, Silvana Pastori, Adele Tono, Barbara Rovelli, Eleonora Gasparotto | Presidio Polispecialistico di Mariano Comense - ASST Lariana | SS InterDipartimentale Diabetologia e Endocrinologia | Mariano Comense (CO) |
| LOMBARDIA | Edoardo Baggio, Maria Elena Lunati, Laura Plebani, Luciana Maria Vallone, Camilla Tinari, Paolo Fiorina | Azienda Socio Sanitaria Fatebenefratelli - Sacco/Ospedale Fatebenefratelli e Oftalmico | SSD Malattie Metaboliche - Diabetologia | Milano (MI) |
| LOMBARDIA | Paola Silvia Anna Morpurgo, Alessandra Gandolfi, Andrea Mario Bolla, Paolo Fiorina | Presidio Ospedaliero Macedonio Melloni - ASST Fatebenefratelli Sacco | Ambulatorio di Diabetologia | MILANO (MI) |
| LOMBARDIA | Ida Pastore, Laura Montefusco, Enrica Chebat, Milena Muratori, Antonio Rossi, Paolo Fiorina, Elisa Lazzaroni | ASST Fatebenefratelli Sacco - Centro Ricerca Invernizzi, Padiglione 62 | Malattie Endocrine e Diabetologia | Milano (MI) |
| LOMBARDIA | Gabriella Piscitelli | ASST Nord Milano | Presidio dei Poliambulatori - Ambulatorio di Piazzale Accursio | Milano (MI) |
| LOMBARDIA | Raffaella Mattioni, Simona Citterio, Nadia De Vincenzo, Rosita Stanga | ASST Nord Milano | Presidio dei Poliambulatori - Ambulatorio di Via Farini | Milano (MI) |
| LOMBARDIA | Marina Scavini, Giuseppe Ancona, Amelia Caretto, Alberto Davalli, Gabriella Galimberti, Andrea Laurenzi, Sabina Martinenghi, Giovanna Petrella, Matteo Rocco Pastore, Erika Pedone, Emanuela Setola, Loredana Bonisolli, Emanuele Bosi, Anna Zanardini, Sonia Letizia, Walter Pepe, Chiara Sartori, Anna Vigna, Valentina Loiacono | Istituto Scientifico-Universitario Ospedale San Raffaele | Diabetologia - UO Medicina Generale ad indirizzo Diabetologico o Endocrino Metabolico | Milano (MI) |

| Region | Authors | Hospital | Department | Town |
|---|---|---|---|---|
| LOMBARDIA | Alessandro Saibene, Adolfo Bianchi, Carlo Augusto Lovagnini Scher, Marco Laneri, Stefano Benedini, Regina Dagani, Elena Mion | Clinica Polispecialistica San Carlo S.r.l. | Sevizio di Diabetologia | Paderno Dugnano (MI) |
| LOMBARDIA | Giosuè Ghilardi, Santo Furneri | ASST Bergamo Est - Ospedale M.O. Locatelli | Servizio di Diabetologia | Piario (BG) |
| LOMBARDIA | Valeria Valdes, Marica Sormani, Daniela Carugo, Michela Gianni, Antonio Tempesta, Anna Maria Schiatti, Angelina D'Andrea | Presidio Ospedaliero di Rho - ASST Rhodense | Ambulatorio di Diabetologia | Rho (MI) |
| LOMBARDIA | Ida Mangone, Veronica Vilei | ASST della Brianza | SSD di Malattie Endocrine, del Ricambio e della Nutrizione | Seregno (MB) |
| LOMBARDIA | Cesare Berra, Daniele Cannavaro, Laura Folini, Caterina Conte, Roberto Manfrini | IRCCS Multimedica | UO di Diabetologia e Malattie Metaboliche | Sesto San Giovanni (MI) |
| LOMBARDIA | Giancarla Meregalli, Denise Berzi, Franco Forloni, Francesco Indovina, Francesca Nicolì, Francesca Viglino, Giovanni Vitali, Giulia Ongis | ASST Bergamo Ovest | SSD Malattie Endocrine - Centro di riferimento regionale per il diabete | Treviglio (BG) |
| LOMBARDIA | Stefano Fazion, Giovanni Rignanese, Gigliola Botta, Federica Chiari, Simona Rossi, Marina Scaravelli | ASST-Mantova, Ambulatorio di Viadana | SSD di Diabetologia e Malattie Metaboliche | Viadana (MN) |
| LOMBARDIA | Giorgia Dito, Marta Di Stefano, Silvia Galliani, Roberto Roncoroni, Ida Mangone | ASST della Brianza | SSD di Malattie Endocrine, del Ricambio e della Nutrizione | Vimercate (MB) |
| MARCHE | Antonio Iannilli, Valentina Tiberi, Monica Marino, Antonia Capogna, Sara Santarelli, Valentino Cherubini | Azienda Ospedaliero-Universitaria Ospedali Riuniti Ancona - Presidio G. Salesi | SOD Diabetologia Pediatrica | Ancona (AN) |
| MARCHE | Massimiliano Petrelli, Francesca Silvetti, Alessio Pieroni, Giorgia Squartini, Lara Giovannini, Carlotta Peroni, Lucia Stella, Michele Perrone | Ospedali Riuniti di Ancona | Clinica di Endocrinologia e Malattie del Metabolismo | Ancona (AN) |
| MARCHE | Elena Tortato, Federica Turchi, Maria Paola Luconi, Manuel Lagonigro | INRCA - IRCCS | UOC Malattie Metaboliche e Diabetologia | Ancona (AN) |
| MARCHE | Rosa Anna Rabini, Graziano Simonella, Milena Santangelo, Marianna Galetta, Federica D'Angelo | AST di Ascoli Piceno - Ospedale Mazzoni | UOC di Malattie Metaboliche e Diabetologia | Ascoli Piceno (AP) |
| MARCHE | Natalia Busciantella Ricci, Michelina Radatti, Giacomo Maccagnani, Rachele Gentile | Ospedale Santa Maria della Pietà Camerino-AST Macerata | UOSD Diabetologia Camerino | Camerino (MC) |

| Region | Authors | Hospital | Department | Town |
|---|---|---|---|---|
| MARCHE | Cristian Quattrini, Clara Alessiani | AST MC - Distretto di Civitanova Marche | UOSD Diabetologia | Civitanova Marche (MC) |
| MARCHE | Vanessa Ronconi, Giorgio Montecchiani, Aurora Pirani, Michela Mariotti, Maria Teresa Valeri, Valentina Marinelli | AST Ancona | UOSD Diabetologia e Malattie Metaboliche | Fabriano (AN) |
| MARCHE | Gabriella Garrapa, Anna Cani, Erica Landini, Giulio Lucarelli, Manuela Montoni, Isabella Occhialini, Lara Riccialdelli, Carla Spendolini | Ospedale Santa Croce-Fano- AST Pesaro Urbino | UOSD Endocrinologia e Diabetologia | Fano (PU) |
| MARCHE | Paola Pantanetti, Grazia Michetti, Sandra Di Marco, Giovanni Cangelosi, Cristina De Carolis, Marco Di Giacinti, Orietta Pazzi, Gianluca Cerasoli, Silvia Coacci, Nadia Francucci | PO A. Murri - AST Fermo | UOSD Diabetologia | Fermo (FM) |
| MARCHE | Paola Canibus, Franco Gregorio, Marina Cardinaletti, Francesca Paggi, Roberta Bruschi, Rossella Cittadini, Samantha Bucci | AST Ancona - Jesi | UOSD Malattie Metaboliche e Diabetologia | Jesi (AN) |
| MARCHE | Francesca Giampaoli, Manuela Ferroni, Anna Maria Frascati, Francesca Carletti, Martina Mengani, Gabriele Brandoni | Ospedale Santa Casa - Loreto AST Ancona | Diabetologia | Loreto (AN) |
| MARCHE | Marilena Giovagnetti, Barbara Polenta, Francesca Borroni, Vincenzo Carletti, Samuela Lardelli, Francesca Carletti, Andrea Benigni, Ilaria Peretti, Anna Maria Tesei, Jihane Lagtaa, Paola Lattanzi, Gabriele Brandoni | AST Macerata Presidio Ospedaliero Macerata | Diabetologia | Macerata (MC) |
| MARCHE | Luigi Maggiulli, Giulio Lucarelli, Alessandro Micillo, Anna Cani, Francesca Felicita, Antonio Russo, Milena Giusti, Fabiola Lizzadro, Giuseppina Fiori, Milena Paris, Antonella Sanchini | Ospedale San Salvatore, AST Pesaro - Urbino | UOSD Endocrinologia e Diabetologia | Pesaro (PU) |
| MARCHE | Maria Zecchini, Barbara Arpiani, Tonia Lattanzi, Andrea Benigni, Ilaria Peretti, Francesca Carletti, Gabriele Brandoni | Ospedale Santa Lucia - Recanati AST Macerata | Diabetologia | Recanati (MC) |
| MARCHE | Rosa Anna Rabini, Marianna Galetta, Milena Santangelo, Federica D'Angelo, Graziano Simonella | AST di Ascoli Piceno - Ospedale Madonna Del Soccorso | UOC Malattie Metaboliche e Diabetologia | San Benedetto del Tronto (AP) |
| MARCHE | Marilena Giovagnetti, Barbara Polenta, Samuela Lardelli, Valentina Maccari, Elisabetta Paperi, Cecilia Losito, Katiuscia Bonifazi, Erica Canzonetta, Paola Molinari, Laura Sabbatini, Gianina Nitu, Erika Bianconi, Gabriele Brandoni | Presidio Ospedaliero San Severino Marche - AST Macerata | Reparto Diabetologia | San Severino Marche (MC) |
| MARCHE | Silvia Rilli, Francesca Paggi, Luigi Lanari, Gessica Tinti, Samantha Bucci | AST Ancona - Senigallia | UOSD Diabetologia Nutrizione Clinica | Senigallia (AN) |

| Region | Authors | Hospital | Department | Town |
|---|---|---|---|---|
| MARCHE | Maurizio Sudano, Claudio Molaioni, Maria Assunta Carlucci | AST Pesaro-Urbino | UOSD Diabetologia e Malattie Metaboliche - Urbino | Urbino (PU) |
| MOLISE | Mariarosaria Cristofaro, Simonetta Di Vincenzo | A.S.R.e.M. Presidio Ospedaliero A. Cardarelli | SC Endocrinologia - Diabetologia - Malattie Metaboliche | Campobasso (CB) |
| PIEMONTE | Emanuele Fraticelli, Elisabetta Benedusi, Andi Masha, Maria Elena Valera Mora, Leonardo Di Martino, Giovanni La Motta, Teresa D'Ambrosio, Margherita Fissore, Manuela Morello, Cristiana Olivero, Loredana Silvosi, Paola Viberti, Cristina Peirano, Marco Pusceddu | ASL CN02 Alba Bra | SSD Endocrinologia, Diabetologia e Malattie Metaboliche- Sede di Alba | Alba (CN) |
| PIEMONTE | Marco Gallo, Enrico Gabellieri, Giulia Bendotti, Emilia Biamonte, Paola Leporati, Alberto Ragni | AOU SS Antonio e Biagio e Cesare Arrigo | SC Endocrinologia e Malattie Metaboliche | Alessandria (AL) |
| PIEMONTE | Maria Chantal Ponziani, Chiara Bima, Piero Radaelli, Irene Samperi, Mara Steffanini, Marinella Platini, Isabella Simonetta Bagatin, Laura Cerini, Raffaella Molinari, Barbara Patrucco, Giovanna Ziero, Antonello Leo | Ospedale SS Trinità - ASL Novara | SSV Dipartimento di Malattie Metaboliche e Diabetologia | Borgomanero (NO) |
| PIEMONTE | Emanuele Fraticelli, Elisabetta Benedusi, Andi Masha, Maria Elena Valera Mora, Leonardo Di Martino, Giovanni La Motta, Teresa D'Ambrosio, Margherita Fissore, Manuela Morello, Cristiana Olivero, Loredana Silvosi, Paola Viberti, Cristina Peirano, Marco Pusceddu | ASL CN2 Alba Bra | SSD Endocrinologia, Diabetologia e Malattie Metaboliche- Sede di Bra | Bra (CN) |
| PIEMONTE | Emanuele Fraticelli, Elisabetta Benedusi, Andi Masha, Maria Elena Valera Mora, Leonardo Di Martino, Govanni La Motta, Teresa D'Ambrosio, Margherita Fissore, Manuela Morello, Cristiana Olivero, Loredana Silvosi, Paola Viberti, Cristina Peirano, Marco Pusceddu | ASL CN2 Alba Bra | SSD Endocrinologia, Diabetologia e Malattie Metaboliche- Sede di Canale | Canale (CN) |
| PIEMONTE | Francesco Romeo, Giuliana Micali, Giovanna Saraceno, Dolores Marzano, Loredana Cuccia, Massimo Rainero, Sandra Vendemiati, Antonela Bursuc, Liliana Audero, Mirella Fornero, Marella Doglio | Ospedale San Lorenzo - ASL TO5 | SC Diabetologia Territoriale | Carmagnola (TO) |
| PIEMONTE | Francesco Romeo, Giovanna Saraceno, Carla Origlia, Loredana Cuccia, Dolores Marzano, Elisa Nada, Patrizia Giai Via, Cassandra Roma, Marisol Ibet Cherigo, Mariangela Incani, Simona Chiara, Marella Doglio, Barbara Tartaglino, Taisir Mahagna | Ospedale Maggiore di Chieri | SC Diabetologia Territoriale | Chieri (TO) |
| PIEMONTE | Riccardo Fornengo, Valentina Gatto, Lidia Di Vito, Elisa Marinazzo, Maria Divina Pascuzzo, Stefano Allasia, Marina Valenzano | ASLTO4 Distretto di Chivasso e San Mauro e Distretto di Settimo Torinese | SSD Diabetologia | Chivasso (TO) |

| Region | Authors | Hospital | Department | Town |
|---|---|---|---|---|
| PIEMONTE | Francesco Caraffa, Daniela Gallo, Riccardo Fornengo | ASLTO4 Distretto di Ciriè | SSD di Diabetologia | Ciriè (TO) |
| PIEMONTE | Francesco Tassone, Claudia Baffoni, Micaela Pellegrino, Federico Arecco, Elena Castellano, Andrea Craparo, Chiara Bona, Anna Pia | ASO S. Croce e Carle Di Cuneo | Endocrinologia e Diabetologia | Cuneo (CN) |
| PIEMONTE | Giuseppe Placentino, Maura Rinaldi, Monica Adriana Resnik Scalella, Erika Mangione | Ospedale S. Biagio | SOC di Diabetologia e Malattie Metaboliche | Domodossola (VB) |
| PIEMONTE | Riccardo Fornengo, Maria Divina Pascuzzo, Stefano Allasia, Elisa Marinazzo | ASLTO4 Distretto di Ivrea e Distretto di Cuorgne | SSD Diabetologia | Ivrea (TO) |
| PIEMONTE | Francesco Romeo, Roberta Manti, Andrea Corino, Taisir Mahagna, Massimo Rainero, Cristina Linzalata, Alessandra Fontana, Cristina Laiolo, Rosa Mortello, Ornella Boscolo, Florina Radulescu, Anna Paola Quaranta, Paola Chiaberti, Marella Doglio | Distretto Sanitario ASL TO5 sede di Moncalieri | SC Diabetologia Territoriale | Moncalieri (TO) |
| PIEMONTE | Francesco Romeo, Loredana Brocato, Giovanna Saraceno, Andrea Corino, Barbara Sieve, Sandra Fasano, Filomena D'Angelo, Concetta Franchina, Simonetta Broggio, Massimo Rainero, Cristina Linzalata, Marella Doglio, Maria Teresa Dirella, Taisir Mahagna | Ospedale Maggiore - ASL TO5 | Diabetologia e Malattie Metaboliche sede di Nichelino | Nichelino (TO) |
| PIEMONTE | Silvia Maria Abate, Marica Pinna, Rosetta Popolizio, Elena Maria Voglino, Mirella Scarampi, Mauro Stroppiana, Deborah Navone, Susanna Baldi, Mirella Andaloro, Patrizia Colombano, Lorella Tibaldi, Maria Bongiorno | ASL AT - Asti | Medicina Polifunzionale | Nizza Monferrato (AT) |
| PIEMONTE | Gianluca Aimaretti, Paolo Marzullo, Flavia Prodam, Marina Caputo, Tommaso Daffara, Marco Zavattaro, Maria Grazia Mauri, Alice Ferrero | A.O.U. Maggiore della Carita' Novara | SCDU Endocrinologia | Novara (NO) |
| PIEMONTE | Giuseppe Placentino, Maura Rinaldi, Monica Adriana Resnik Scalella, Erika Mangione | ASL Verbania - Cusio - Ossola (VCO) Piemonte | SSD Diabetologia e Malattie Metaboliche | Omegna (VB) |
| PIEMONTE | Enrico Pergolizzi, Anna Rosa Bogazzi, Giovanna Bendinelli | Ospedale di Pianezza ASL TO3 | SSD Malattie Endocrine e Diabetologia | Pianezza (TO) |
| PIEMONTE | Enrico Pergolizzi, Paola Gennari, Daniela Gaia, Giuliana Micali, Patrizia Marino, Katia Bonomo, Elisabetta Rossetto, Tiziana Stefani, Antonio Modarelli, Laura Bellino | Ospedale Civile di Pinerolo ASL TO3 | SSD Malattie Endocrine e Diabetologia | Pinerolo (TO) |
| PIEMONTE | Rita Graziella Guarnieri, Luisa Barana, Emanuela Candida Massimetti | ASL 12 Biella - Ospedale Degli Infermi | Diabetologia e Endocrinologia | Ponderano (BI) |

| Region | Authors | Hospital | Department | Town |
|---|---|---|---|---|
| PIEMONTE | Enrico Pergolizzi, Anna Rosa Bogazzi, Giovanna Bendinelli, Domenica Giuffrida, Marta Aventaggiato, Elisa Me | Ospedale di Rivoli ASL TO3 | SSD Malattie Endocrine e Diabetologia | Rivoli (TO) |
| PIEMONTE | Enrico Pergolizzi, Maria Ausilia Caccavale, Mariantonietta Secchi | Ospedale Civile di Susa ASL TO3 | SSD Malattie Endocrine e Diabetologia | Susa (TO) |
| PIEMONTE | Maria Antonietta Altea, Marcella Libera Balbo, Maria Pia Norma Bavoso, Alberto Blatto, Francesco Brogna, Luciella Cirinnà, Alessandra Clerico, Ginevra Corneli, Claudia De Natale, Fabiana Di Noi, Francesca Garino, Carlotta Gauna, Cristina Gottero, Elena Gramaglia, Michele Iaccarino, Linda Leonardi, Annarita Leoncavallo, Alessandra Rita Mainolfi, Lisa Marafetti, Cristina Matteoda, Enzo Megale, Salvatore Endrio Oleandri, Maria Divina Pascuzzo, Giuliana Petraroli, Valentina Ramella Gigliardi, Claudio Rossi, Silvia Rovere, Daniela Sansone, Maria Angela Seardo, Claudia Toscano, Felicia Visconti, Nadia Bonelli, Daniela Rosso | ASL Città di Torino - Ospedale Maria Vittoria | SC Endocrinologia Malattie Metaboliche | Torino (TO) |
| PIEMONTE | Antonietta Altea Maria, Marcella Libera Balbo, Maria Pia Norma Bavoso, Alberto Blatto, Francesco Brogna, Luciella Cirinnà, Alessandra Clerico, Ginevra Corneli, Claudia De Natale, Nadia Bonelli, Fabiana Di Noi, Francesca Garino, Carlotta Gauna, Cristina Gottero, Elena Gramaglia, Michele Iaccarino, Linda Leonardi, Annarita Leoncavallo, Alessandra Rita Mainolfi, Lisa Marafetti, Cristina Matteoda, Enzo Megale, Salvatore Endrio Oleandri, Maria Divina Pascuzzo, Giuliana Petraroli, Valentina Ramella Gigliardi, Claudio Rossi, Silvia Rovere, Daniela Sansone, Maria Angela Seardo, Claudia Toscano, Felicia Visconti, Daniela Rosso | ASL Città di Torino - Presidio Ospedaliero Oftalmico | SC Endocrinologia e Malattie Metaboliche | Torino (TO) |
| PIEMONTE | Maria Antonietta Altea, Marcella Libera Balbo Balbo, Maria Pia Norma Bavoso, Alberto Blatto, Francesco Brogna, Nadia Bonelli, Luciella Cirinnà, Alessandra Clerico, Ginevra Corneli, Claudia De Natale, Fabiana Di Noi, Francesca Garino, Carlotta Gauna, Cristina Gottero, Elena Gramaglia, Michele Iaccarino, Linda Leonardi, Annarita Leoncavallo, Alessandra Rita Mainolfi, Lisa Marafetti, Cristina Matteoda, Enzo Megale, Salvatore Endrio Oleandri, Maria Divina Pascuzzo, Giuliana Petraroli, Valentina Ramella Gigliardi, Claudio Rossi, Silvia Rovere, Daniela Sansone, Maria Angela Seardo, Claudia Toscano, Felicia Visconti, Daniela Rosso | ASL Città di Torino | Poliambulatorio C.so Toscana | Torino (TO) |
| PIEMONTE | Maria Antonietta Altea, Marcella Libera Balbo Balbo, Maria Pia Norma Bavoso, Alberto Blatto, Francesco Brogna, Nadia Bonelli, Luciella Cirinnà, Alessandra Clerico, Ginevra Corneli, Claudia De Natale, Fabiana Di Noi, Francesca Garino, Carlotta Gauna, Cristina Gottero, Elena Gramaglia, Michele | ASL Città di Torino | Polimabulatorio Lungo Dora Savona | Torino (TO) |

| Region | Authors | Hospital | Department | Town |
|---|---|---|---|---|
| | Iaccarino, Linda Leonardi, Annarita Leoncavallo, Alessandra Rita Mainolfi, Lisa Marafetti, Cristina Matteoda, Enzo Megale, Salvatore Endrio Oleandri, Maria Divina Pascuzzo, Giuliana Petraroli, Valentina Ramella Gigliardi, Claudio Rossi, Silvia Rovere, Daniela Sansone, Maria Angela Seardo, Claudia Toscano, Felicia Visconti, Daniela Rosso | | | |
| PIEMONTE | Maria Antonietta Altea, Marcella Libera Balbo Balbo, Maria Pia Norma Bavoso, Alberto Blatto, Francesco Brogna, Nadia Bonelli, Luciella Cirinnà, Alessandra Clerico, Ginevra Corneli, Claudia De Natale, Fabiana Di Noi, Francesca Garino, Carlotta Gauna, Cristina Gottero, Elena Gramaglia, Michele Iaccarino, Linda Leonardi, Annarita Leoncavallo, Alessandra Rita Mainolfi, Lisa Marafetti, Cristina Matteoda, Enzo Megale, Salvatore Endrio Oleandri, Maria Divina Pascuzzo, Giuliana Petraroli, Valentina Ramella Gigliardi, Claudio Rossi, Silvia Rovere, Daniela Sansone, Maria Angela Seardo, Claudia Toscano, Felicia Visconti, Daniela Rosso | ASL Città di Torino | Poliambulatorio Montanaro | Torino (TO) |
| PIEMONTE | Maria Antonietta Altea, Marcella Libera Balbo Balbo, Maria Pia Norma Bavoso, Alberto Blatto, Francesco Brogna, Nadia Bonelli, Luciella Cirinnà, Alessandra Clerico, Ginevra Corneli, Claudia De Natale, Fabiana Di Noi, Francesca Garino, Carlotta Gauna, Cristina Gottero, Elena Gramaglia, Michele Iaccarino, Linda Leonardi, Annarita Leoncavallo, Alessandra Rita Mainolfi, Lisa Marafetti, Cristina Matteoda, Enzo Megale, Salvatore Endrio Oleandri, Maria Divina Pascuzzo, Giuliana Petraroli, Valentina Ramella Gigliardi, Claudio Rossi, Silvia Rovere, Daniela Sansone, Maria Angela Seardo, Claudia Toscano, Felicia Visconti, Daniela Rosso | ASL Città di Torino | Poliambulatorio Pacchiotti | Torino (TO) |
| PIEMONTE | Maria Antonietta Altea, Marcella Libera Balbo Balbo, Maria Pia Norma Bavoso, Alberto Blatto, Francesco Brogna, Nadia Bonelli, Luciella Cirinnà, Alessandra Clerico, Ginevra Corneli, Claudia De Natale, Fabiana Di Noi, Francesca Garino, Carlotta Gauna, Cristina Gottero, Elena Gramaglia, Michele Iaccarino, Linda Leonardi, Annarita Leoncavallo, Alessandra Rita Mainolfi, Lisa Marafetti, Cristina Matteoda, Enzo Megale, Salvatore Endrio Oleandri, Maria Divina Pascuzzo, Giuliana Petraroli, Valentina Ramella Gigliardi, Claudio Rossi, Silvia Rovere, Daniela Sansone, Maria Angela Seardo, Claudia Toscano, Felicia Visconti, Daniela Rosso | ASL Città di Torino | Poliambulatorio Torino Gorizia | Torino (TO) |
| PIEMONTE | Maria Antonietta Altea, Marcella Libera Balbo Balbo, Maria Pia Norma Bavoso, Alberto Blatto, Francesco Brogna, Nadia Bonelli, Luciella Cirinnà, Alessandra Clerico, Ginevra Corneli, Claudia De Natale, Fabiana Di Noi, Francesca Garino, Carlotta Gauna, | ASL Città di Torino | Poliambulatorio Torino Monginevro | Torino (TO) |

| Region | Authors | Hospital | Department | Town |
|---|---|---|---|---|
| | Cristina Gottero, Elena Gramaglia, Michele Iaccarino, Linda Leonardi, Annarita Leoncavallo, Alessandra Rita Mainolfi, Lisa Marafetti, Cristina Matteoda, Enzo Megale, Salvatore Endrio Oleandri, Maria Divina Pascuzzo, Giuliana Petraroli, Valentina Ramella Gigliardi, Claudio Rossi, Silvia Rovere, Daniela Sansone, Maria Angela Seardo, Felicia Toscano, Felicia Visconti, Daniela Rosso | | | |
| PIEMONTE | Enrico Pergolizzi, Anna Rosa Bogazzi, Giovanna Bendinelli | Ospedale di Venaria Reale ASL TO3 | SSD Malattie Endocrine e Diabetologia | Venaria Reale (TO) |
| PIEMONTE | Giuseppe Placentino, Maura Rinaldi, Monica Adriana Resnik Scalella, Erika Mangione | Ospedale di Verbania | SOC di Diabetologia e Malattie Metaboliche | Verbania (VB) |
| PIEMONTE | Roberta Paltro, Simona Bandinelli, Arianna Busti, Valeria Cambria, Ilaria Leone | ASL VC | SS Diabetologia e Malattie Metaboliche | Vercelli (VC) |
| PUGLIA | Alessandra Di Flaviani, Saverio Fatone, Federica Giampetruzzi | ACISMOM Andria | Centro Diabetologico | Andria (BT) |
| PUGLIA | Francesco Giorgino, Luigi Laviola, Gian Pio Sorice | Azienda Ospedaliero Universitaria Consorziale Policlinico Bari | Endocrinologia Universitaria | Bari (BA) |
| PUGLIA | Olga Lamacchia, Stefania Annese, Ester Laudadio, Matteo Paradiso | OORR Riuniti di Foggia | Endocrinologia | Foggia (FG) |
| PUGLIA | Fabrizio Diacono | ASL Lecce - Poliambulatorio Martano | Ambulatorio di diabetologia | Martano (LE) |
| PUGLIA | Irene Alemanno, Cristiana Lattanzio | ASL Lecce DSS Poggiardo | Endocrinologia | Poggiardo (LE) |
| PUGLIA | Salvatore De Cosmo, Anna Rauseo, Pamela Piscitelli | IRCCS Casa Sollievo della Sofferenza | Medicina Interna - Endocrinologia | San Giovanni Rotondo (FG) |
| PUGLIA | Giuliana Cazzetta, Maria Teresa Branca, Marzia Filesi | ASL Lecce | Poliambulatorio Tricase | Tricase (LE) |
| PUGLIA | Giuliana Cazzetta, Claudia Toma | ASL Lecce | Poliambulatorio Distrettuale Ugento | Ugento (LE) |
| SARDEGNA | Luisa Porcu, Giovanna Paola Tolu, Francesca Zanda, Giuseppa Aguglia, Maria Pasqua Piras, Carla Verdi, Serafina Sini, Rosanna Salvatore | ASL-Sassari - SS Diabetologia - Distretto di Alghero | SS Diabetologia | Alghero (SS) |
| SARDEGNA | Roberto Seguro, Francesca Scano, Valentina Maria Cambuli, Anna Rita Angioni | Ospedale San Michele Azienda Ospedaliera G. Brotzu | Struttura Complessa di Diabetologia | Cagliari (CA) |

| Region | Authors | Hospital | Department | Town |
| --- | --- | --- | --- | --- |
| SARDEGNA | Francesca Spanu, Rosangela Maria Pilosu, Paola Pisanu, Maria Carla Farci, Antonietta Casu, Adelina Porru, Maria Vitalia Ortu, Maria Margherita Gessa, Carlotta Macis, Giorgetta Farina, Maria Pina Puddu | Presidio San Giovanni di Dio | Diabetologia e malattie metaboliche | Cagliari (CA) |
| SARDEGNA | Giacomo Guaita, Chiara Serafini, Paola Elisa Meloni, Michele Fosci, Sandro Cocco, Nada Richterova, Sergio Corsini, Loredana Marongiu, Assunta Poggi, Antonella Vanni | Presidio Ospedaliero Sirai ASL Sulcis Iglesiente | UOC Diabetologia, Endocrinologia e Malattie Metaboliche | Carbonia (SU) |
| SARDEGNA | Ilaria Pelligra, Giacomo Guaita, Cinzia Deias, Enzo Tuveri, Anna Maria Cardia, Emiliano Rosina, Graziella Angius, Stefania Leoni, Fanny Marcello, Gianfranco Orrù, Giuseppe Correale | Presidio Ospedaliero Santa Barbara ASL Sulcis Iglesiente | UOC di Diabetologia, Endocrinologia e Malattie Metaboliche | Iglesias (SU) |
| SARDEGNA | Maria Antonietta Fois, Laura Perra, Stefania Deiana | ATS Sardegna ASSL Cagliari - Distretto di Sarcidano - Barbagia di Seulo e Trexenta | Servizio Territoriale Diabetologia di Isili | Isili (CA) |
| SARDEGNA | Gisella Meloni, Maria Grazia Pani, Claudia Putzu, Paola Elisa Meloni, Sestilia Cozzolino, Ornella Casula, Liliana Cerina, Andrea Cabiddu, Gilda Luisa Usala | ATS Sardegna - ASSL Lanusei | UO Diabetologia e Malattie Metaboliche | Lanusei (NU) |
| SARDEGNA | Alessia Prinzis, Giuseppina Gattu | ASSL Nuoro | Poliambulatorio Macomer - Diabetologia | Macomer (NU) |
| SARDEGNA | Francesca Spanu, Elena Loy, Marta Tuveri, Diego Mastino, Assunta Stella Belgiorno, Giovanna Fabiana Zedda, Silvana Serra, Manuela Cauli, Nisella Murtas, Efisio Cossu | Azienda Ospedaliera Universitaria di Cagliari - Policlinico D. Casula | SSD Diabetologia | Monserrato (CA) |
| SARDEGNA | Alfonso Gigante, Paolo Giuseppe Michele Bianco, Rosalia Polo, Luisa Sollai, Sestilia Cozzolino, Talia Serra, Liliana Marteddu, Bastianina Pira, Antonella Ruiu, Daniela Salaris | Ospedale C. Zonchello | SC di Diabetologia | Nuoro (NU) |
| SARDEGNA | Giancarlo Tonolo, Maria Filippina Angius, Rosanna Vacca, Ilario Carta, Sara Cherchi, Lucia Canu, Maura Serena Ledda, Angela Becciu, Giuliana Giorgi, Gianpiera Amadori, Adriana Ventroni, Maria Antonietta Taras, Paola Rubbiani, Maria Porceddu, Rita Impagliazzo, Elvira Terrazzoni | ASL 2 Olbia - PO San Giovanni di Dio | SC Aziendale Diabetologia | Olbia (OT) |
| SARDEGNA | Concetta Clausi, Luisa Sechi, Maria Maddalena Atzeni, Giuseppina Frau, Marco Mastinu, Monica Obinu, Giuseppina Melis | ASL Oristano - Ospedale San Martino | UO Malattie Metaboliche e Diabetologia | Oristano (SS) |
| SARDEGNA | Maria Antonietta Cambosu | ASL Nuoro | Polimabulatorio - Distretto di Siniscola - Orosei | Orosei (NU) |

| Region | Authors | Hospital | Department | Town |
|---|---|---|---|---|
| SARDEGNA | Marco Mameli | Distretto Sanitario di Sassari - ASSL Sassari | Servizio di Diabetologia - Poliambulatorio di Porto Torres | Porto Torres (SS) |
| SARDEGNA | Fernando Farci, Mariangela Ghiani, Rossella Cau, Danila Pistis, Maurizio Sparano, Loredanna Piras, Ilaria Loddo, Maria Maddalena Pitturru, Annalisa Cogoni, Evelin Pandolfi, Valeria Tavolacci, Angela Podda | ATS Sardegna - ASSL Cagliari | UO Diabetologia Distretto di Quartu - Parteolla | Quartu Sant'Elena (CA) |
| SARDEGNA | Raffaella Derai, Martina Melis, Chiara Satta, Marina Armeni, Stefania Casula, Cira Lombardo, Maria Antonietta Pisano, Riccarda Garau, Maria Rosaria Meloni, Elisabetta Cossu, Simonetta Mamusa | ASL Medio Campidano - Poliambulatorio Sanluri | UOC Diabetologia | Sanluri (SU) |
| SARDEGNA | Alessandro Gentilini, Pietro Fresu, Carboni Antonello, Margherita Idda, Alberto Manconi, Tiziana Congiu, Angelo Fancellu, Maristella Masala | ASSL1 Sassari- Distretto Di Sassari | SC di Diabetologia e Malattie Del Metabolismo | Sassari (SS) |
| SARDEGNA | Alessio Lai, Stefania Deiana, Tiziana Mudadu | ASSL Cagliari | Ambulatorio di Diabetologia - Poliambulatori di Senorbi | Senorbi (CA) |
| SARDEGNA | Maria Antonietta Cambosu | Poliambulatorio di Siniscola ASL Nuoro | Poliambulatorio di Diabetologia | Siniscola (NU) |
| SARDEGNA | Jacopo Salvatore Bulla, Giuseppina Gattu, Alfonso Gigante | ASL 3 Nuoro - Distretto Sanitario Di Sorgono - Ospedale San Camillo | Servizio Diabetologia | Sorgono (NU) |
| SARDEGNA | Fabrizia Caucci | Distretto di Sassari | Poliambulatorio San Camillo - Diabetologia | Sorso - Sassari (SS) |
| SARDEGNA | Maria Chiara Cocco, Anna Rita Fanni, Adalgisa Boi, Tatiana Lai | Distretto Sanitario di Sarrabus Gerrei ASL 8 Cagliari | Ambulatorio Diabetologia Villasimius | Villasimius (CA) |
| SICILIA | Lucia Frittitta, Teresa Ballirò, Roberto Baratta, Agostino Milluzzo, Laura Sciacca | ARNAS Garibaldi P.O. Garibaldi Nesima | UOSD Centro Antidiabetico e cura dell'obesità | Catania (CT) |
| SICILIA | Concetta Gatta, Caterina Merendino | A.O. Universitaria - Policlinico Vittorio Emanuele di Catania | I Divisione di Medicina Presso Presidio Vittorio Emanuele | Catania (CT) |
| SICILIA | Antonietta Maria Scarpitta, Antonino Lo Presti | P.O. Paolo Borsellino Marsala - A.S.P. Trapani | UOC di Diabetologia e Malattie Metaboliche | Marsala (TP) |
| SICILIA | Giuseppina Russo, Annalisa Giandalia, Domenica Ruggeri, Cristina Quartarone | A.O.U. Policlinico Universitario G. Martino | UOC Medicina Interna - Malattie Metaboliche | Messina (ME) |

| Region | Authors | Hospital | Department | Town |
|---|---|---|---|---|
| SICILIA | Giuseppe Mattina | PTA Biondo | Ambulatorio di Diabetologia | Palermo (PA) |
| SICILIA | Giuseppe Mattina | PTA Palermo Centro | Poliambulatorio di Diabetologia | Palermo (PA) |
| SICILIA | Vittoria Sesta, Franca Daidone | ASP 8 Siracusa Ospedale Rizza | Poliambulatorio di Diabetologia | Siracusa (SR) |
| TOSCANA | Fabio Baccetti, Isabella Crisci Crisci, Giovanna Gregori, Mary Mori | Centro Polispecialistico Monterosso | SSD Diabetologia | Carrara (MS) |
| TOSCANA | Paola Orsini, Daniela Cannistraro, Laura Russo | Ospedale Cecina | Diabetologia | Cecina (LI) |
| TOSCANA | Laura Sambuco, Loredana Rizzo, Giovanni De Gennaro, Walter Baronti | Azienda USL Toscana Sud Est P.O. Misericordia | UOC Diabetologia | Grosseto (GR) |
| TOSCANA | Giuseppe Viccica, Margherita Occhipinti, Stefania Bertoli, Sabrina Cosimi | AUSL Toscana Nord Ovest - Ospedale Versilia | Reparto di Diabetologia | Lido di Camaiore (LU) |
| TOSCANA | Paola Orsini, Emilia Lacaria, Francesca Pancani, Valentina Verdiani, Graziano Di Cianni | ASL Toscana Nord Ovest | UOC Diabetologia | Livorno (LI) |
| TOSCANA | Alberto Di Carlo, Agnese Biagini, Ilaria Casadidio, Ilaria Cuccuru, Cristina Lencioni | Ospedale Campo di Marte - AUSL Toscana Nord Ovest | Diabetologia e Malattie Metaboliche | Lucca (LU) |
| TOSCANA | Chiara Caiulo, Alice Valeria Magiar, Elisa Giurgola, Secondina Viti, Irene Sara Howard, Alessia Lazzarini, Milva Lazzeretti, Angela Pellegrini, Andrea Mancini, Roberto Anichini | Ospedale Cosma Damiano | UO Diabetologia Area Pistoiese | Pescia (PT) |
| TOSCANA | Valerio Gherardini | Ospedale Villa Marina di Piombino - USL 6 Livorno | Sezione Diabetologia - UO Medicina Interna | Piombino (LI) |
| TOSCANA | Roberto Anichini, Claudia Cosentino, Elisabetta Salutini, Anna Tedeschi, Chiara Delli Poggi, Marco Perini, Lisetta Butelli, Cinzia Perini, Rossella Picciafuochi | Ospedale San Jacopo | UO Diabetologia Area Pistoiese | Pistoia (PT) |
| TOSCANA | Paola Orsini, Giovanna Villani, Paolo Francesco Passannanti | Ospedale di Portoferraio | Reparto di Diabetologia | Portoferraio (LI) |
| TOSCANA | Maria Calabrese, Caterina Lamanna, Marta Seghieri, Lucia Ianni, Monica Lorenzetti, Angela Marsocci, Sandra Guizzotti, Ylenia Vignoli, Pamela Luccarini, Graziella Scarcella, Carmelina Amendola, Ahoua Sagnon | Presidio Misericordia e Dolce - USL Toscana Centro | UOSD Diabetologia | Prato (PO) |

| Region | Authors | Hospital | Department | Town |
|---|---|---|---|---|
| TOSCANA | Giuseppe Viccica, Daniele Sgrò | Ospedale di Volterra | Servizio di Diabetologia | Volterra/Pontedera (PI) |
| TRENTINO ALTO ADIGE | Bruno Fattor, Tiziano Monauni, Dalia Crazzolara, Ilaria Rubbo, Michela Cristini, Marco Dauriz, Endrighi Roberta, Sara Dellandrea | Ospedale Centrale di Bolzano - Ospedale di insegnamento dell'Università Privata di Medicina Paracelso PMU | Servizio di Diabetologia | Bolzano (BZ) |
| TRENTINO ALTO ADIGE | Sandro Inchiostro, Luisella Perina, Stefano Garavelli | APSST Ospedale S. Lorenzo Centro Diabetico di Borgo | Centro Diabetico | Borgo Valsugana (TN) |
| TRENTINO ALTO ADIGE | Susi Martucci | Ospedale di Cavalese | UO Medicina | Cavalese (TN) |
| TRENTINO ALTO ADIGE | Florian Hermann Wöhs, Giampiero Incelli | Ospedale Tappeiner | Servizio Di Diabetologia | Merano (BZ) |
| TRENTINO ALTO ADIGE | Sandro Inchiostro, Luisella Perina, Stefano Garavelli | APSST Ospedale S. Lorenzo - Centro Diabetico Pergine | UO Medicina | Pergine Valsugana (TN) |
| TRENTINO ALTO ADIGE | Sandro Inchiostro, Luisella Perina, Stefano Garavelli | APSST Ospedale S. Lorenzo Centro Diabetico di Primiero | Centro Diabetico | Primiero (TN) |
| TRENTINO ALTO ADIGE | Marlene Dall'Alda, Lorena De Moliner, Mariana Peroni | Ospedale Santa Maria del Carmine di Rovereto | Ambulatorio di Diabetologia | Rovereto (TN) |
| TRENTINO ALTO ADIGE | Massimo Orrasch, Francesca Zambotti, Tiziano Lucianer, Bruna Barcatta, Silvia Clementi, Cristina Faes, Ilaria Nicolao, Roberta Fellin | APSS Trento Ospedale Santa Chiara Poliambulatorio Crosina | Centro Diabetologico | Trento (TN) |
| UMBRIA | Roberto Norgiolini, Anna Marinelli Andreoli, Luigina Biagini, Loredana Calzona, Antonio Mastroianni, Catiuscia Tamburi | Ospedale Civile Città di Castello | SS di Diabetologia | Città Di Castello (PG) |
| UMBRIA | Maria Luisa Picchio, Chiara Pascucci, Paola Lucidi, Francesca Cammilleri, Antonella Monni, Fabio Scarlato, Roberta Trabalza, Sandra Iaquaniello | Centro di Salute Sede di Foligno ASL 2 dell' Umbria | Servizio di Diabetologia | Foligno (PG) |
| UMBRIA | Cecilia Marino, Silvia Arnone, Stefania Venturi, Annarita Petrelli, Antonio Mastroianni, Lucia Fadda | USL Umbria 1 Ospedale Branca di Gubbio | Servizio Diabetologia dell'Alto Chiascio | Gubbio (PG) |
| UMBRIA | Massimo Bracaccia, Marco Tonelli, Alessandro Mechelli | Azienda USL Umbria 2 | UO Diabetologia | Orvieto (TR) |

| Region | Authors | Hospital | Department | Town |
|---|---|---|---|---|
| UMBRIA | Elisabetta Torlone, Carmina Fanelli, Francesca Porcellati, Simone Pampanelli, Gabriele Perriello, Efisio Puxeddu, Elisa Donnini, Valeriana Grassini, Nadia Biccari, Cinzia Minelli, Federica De Carolis, Francesca Mazzasette, Cristiana Vermigli, Giuseppe Murdolo, Raffaella Lupattelli, Maria Teresa Sulpizi, Celeste Ceppitelli | Azienda Ospedaliera Santa Maria della Misericordia | SC Endocrinologia e Malattie del Metabolismo | Perugia (PG) |
| UMBRIA | Paola Del Sindaco, Chiara Di Loreto, Roberta Celleno, Pezzuto Debora, Ambrosi Franca, Silvia Arnone, Laura Piastrella, Marinella Biagini, Silvia Bellavita, Ivonne Ghilardi, Monica Passeri, Tamburi Catiuscia, Luca Barbanera | USL 1 Umbria | UOS Diabetologia Distretto Del Perugino | Perugia (PG) |
| UMBRIA | Carlo Lalli, Francesca Cammilleri, Monia Cintio, Maura Scarponi, Eleonora Carrara, Fabio Scarlato, Luca Sotgiu | Distretto di Spoleto ASL 2 Umbria | Servizio di diabetologia Poliambulatori Distrettuali | Spoleto (PG) |
| UMBRIA | Barbara Fiordiponti, Maria Grazia Massarelli, Carla Cruciani, Maria Bertoldi, Benedetta Carinella, Desiree Multinu, Mara Rita Piacentini, Patrizia Cioli | Azienda USL Umbria 2 | UO Diabetologia | Terni (TR) |
| VALLE D'AOSTA | Giulio Doveri, Emma Lillaz | Ospedale Regionale Umberto Parini | Ambulatori di Medicina-Diabetologia | Aosta (AO) |
| VENETO | Simonetta Lombardi, Silvia Burlina, Dario Cioccoloni, Daniele Raimondo, Giovanni Romanello, Chiara Tommasi, Sabrina Cozza, Isabella Mecenero, Marta Binotto, Andrea Guiotto, Serena Sarti, Eliana Scott | Distretto Est e Ovest Aulss 8 Berica | UOSD Diabetologia ed Endocrinologia Territoriale | Arzignano (VI) |
| VENETO | Alberto Marangoni, Sara Balzano, Rachele Scotton, Maria Ferrari, Alessandro Pianta | Ospedale Bassiano | Centro Antidiabetico | Bassano Del Grappa (VI) |
| VENETO | Corradina Alagona, Massimo Boaretto, Michela Da Rold, Isabella Famà, Sabrina Grisot, Tania Ronzani, Claudia Cibien | ULSS 1 Dolomiti, Presidio Ospedaliero San Martino | UOS Diabetologia | Belluno (BL) |
| VENETO | Cristina Bittante, Lucia Bondesan | ASL 21 CAD Bovolone | Diabetologia | Bovolone (VR) |
| VENETO | Daniela Di Sarra, Monica Benato, Elena Bertoldi, Lara Nicoloso, Marisa Adami | Poliambulatori Ospedale di Bussolengo - AULSS 9 | UO di Medicina | Bussolengo (VR) |
| VENETO | Daniela Di Sarra, Dominga Perrone, Elisabetta Bonvicini, Mara Sartori | ULSS 9 Scaligera - Caprino Veronese | CAD Caprino Veronese | Caprino Veronese (VR) |
| VENETO | Narciso Marin, Alessandra Cosma, Sabrina Battagin, Antonella Maria Di Lucia, Catia Flori, Anna Pisacane, Alessandro Bergamin, Moraika Riggi, Simone Giradin, Laura Nollino, Marialisa Marcon | Ospedale San Giacomo Apostolo - AULSS 2 Marca Trevigiana | UOS di Diabetologia | Castelfranco Veneto (TV) |

| Region | Authors | Hospital | Department | Town |
|---|---|---|---|---|
| VENETO | Andrea Nogara, Viola Sanga, Silvia Di Benedetto | Ospedale Civile di Chioggia ULSS 3 (ex ULSS 14) | UOSD Diabetologia e malattie del Ricambio | Chioggia (VE) |
| VENETO | Vera Frison, Alessio Filippi, Michela Bettio, Anna Coracina, Nino Cristiano Chilelli, Marco Grasso, Giorgia Costantini, Laura Tessarollo, Sabrina Cappellato, Emanuela Agostini, Silvia Convertini, Isabella Zarantonello, Giuliana Taffarello, Felicia Abascia', Lisa Mason, Sandra Settimo | Presidio Ospedaliero di Cittadella, ULSS 6 Euganea | UOSD Diabetologia | Cittadella (PD) |
| VENETO | Laura Nollino | Presidio di Conegliano - ULSS 7 Pieve di Soligo | UOSD Diabetologia | Conegliano (TV) |
| VENETO | Maria Luisa Contin, Angela Pia De Cata, Isabella Negro, Iessica Iacovacci, Nicoletta Panzonato, Valentina Baracco | Ospedale di Dolo | Servizio di Diabetologia | Dolo (VE) |
| VENETO | Ferruccio D'Incau, Anna Altomari, Antonio Volpi | Ospedale S. Maria del Prato - ULSS 2 - Feltre | UOS Diabetologia | Feltre (BL) |
| VENETO | Daniela Di Sarra, Cavicchioli Cecilia, Fiorini Patrizia | Poliambulatori Ospedale Isola - AULSS 9 | CAD Ospedale Isola della Scala | Isola della Scala (VR) |
| VENETO | Federico Bellavere, Carmela Vinci | Ospedale di Jesolo - ASL n.10 Veneto Orientale | UOSD Diabetologia | Jesolo (VE) |
| VENETO | Lucia Bondesan, Cristina Bittante, Giulia Ceradini | ASL0 21 Verona CAD Legnago | Reparto di Medicina | Legnago (VR) |
| VENETO | Simonetta Lombardi, Silvia Burlina, Dario Cioccoloni, Daniele Raimondo, Giovanni Romanello, Chiara Tommasi, Sabrina Cozza, Isabella Mecenero, Marta Binotto, Andrea Guiotto, Serena Sarti, Eliana Scott | Distretto Est e Ovest Aulss 8 Berica | UOSD Diabetologia ed Endocrinologia Territoriale | Lonigo (VI) |
| VENETO | Manuela Moise', Donata Barison, Pierantonio Conton | Distretto 2 ASL 3 Serenissima | Ambulatorio di Diabetologia Distretto 2 | Mestre (VE) |
| VENETO | Michele D'Ambrosio | Ulss 6 Euganea | UOSD Diabetologia | Monselice (PD) |
| VENETO | Michele D'Ambrosio | Ulss 6 Euganea Ospedale di Montagnana | Centro UOSD Diabetologia | Montagnana (PD) |
| VENETO | Narciso Marin, Alessandra Cosma, Elisa Baldasso, Ivana Martini, Maria Zanatta, Lucia Favero, Espartaco Rigo, Laura Nollino, Andrea Dotto, Agostino Paccagnella, Marialisa Marcon | Presidio Ospedaliero di Montebelluna - AULSS 2 Marca Trevigiana | UOS di Diabetologia | Montebelluna (TV) |

| Region | Authors | Hospital | Department | Town |
|---|---|---|---|---|
| VENETO | Luciano Zenari | Ospedale Sacro Cuore Don Calabria | UO di Diabetologia | Negrar (VR) |
| VENETO | Maria Luisa Contin, Loris Bortolato, Michela Dal Pos, Silvia Lunardon, Pierantonio Conton, Oreana Sabatelli, Barbara Centenaro, Palmira Libanore, Martina Aldrigo | USL 13 di Mirano Ospedale di Noale | Servizio di Diabetologia | Noale (VE) |
| VENETO | Alessandra Marques, Lucia Bondesan | ASL 21 CAD Nogara | Reparto di Medicina | Nogara (VR) |
| VENETO | Maria Simoncini, Marco Strazzabosco | Ospedale Milani | Ambulatorio Diabetologico | Noventa Vicentina (VI) |
| VENETO | Angelo Avogaro, Gian Paolo Fadini, Bonora Benedetta Maria, Alberto Maran, Daniela Bruttomesso, Federico Boscari, Mauro Rigato, Monica Vedovato, Cristina Crepaldi, Nicola Vitturi, Andrea Bruttocao, Gabriella Guarneri, Mario Luca Morieri | Azienda Ospedaliera di Padova | Dipartimento di Medicina - UOC Malattie del Metabolismo | Padova (PD) |
| VENETO | Annunziata Lapolla, Giuseppe Bax, Barbara Bonsembiante, Maria Grazia Dalfrà, Alessandra Gallo, Michela Masin, Silvia Minardi, Beatrice Moro, Francesco Piarulli, Isabella Negro, Antonino Pipitone, Giovanni Sartore, Silvia Longhin, Silvia Pastrolin | Università degli Studi di Padova - UO Diabetologia USL 6 Padova | UOC di Diabetologia e Dietetica | Padova (PD) |
| VENETO | | Ospedale di Pieve di Cadore | UO di Medicina - Servizio di Diabetologia | Pieve di Cadore (BL) |
| VENETO | Cristiano Fongher | Casa di Cura Madonna della Salute - ULSS 5 | Ambulatorio di Diabetologia | Porto Viro (RO) |
| VENETO | Isabella Colletti, Milena Zanon, Carmela Vinci | ASL 10 Veneto Orientale Ospedale di Portogruaro | UOSD Diabetologia | Portogruaro (VE) |
| VENETO | Carmela Vinci, Valeria Vallone, Silvia Pinelli | Ospedale S. Donà Di Piave ASL 10 Veneto Orientale | Servizio di Diabetologia | San Donà Di Piave (VE) |
| VENETO | Silvana Costa, Marco Strazzabosco | Ospedale Territoriale di Sandrigo | Ambulatorio Diabetologico | Sandrigo (VI) |
| VENETO | Francesco Calcaterra, Marina Miola, Antonella Senesi, Francesca Dal Molin, Giulia Faccin, Giuliana Maria Mele Bertoldo, Davide Londei | Casa della Salute di Schio | UO Diabetologia Endocrinologia Dietetica | Schio (VI) |
| VENETO | Simonetta Lombardi, Silvia Burlina, Dario Cioccoloni, Daniele Raimondo, Giovanni Romanello, Chiara Tommasi, Sabrina Cozza, Isabella Mecenero, Marta Binotto, Andrea Guiotto, Serena Sarti, Eliana Scott | Distretto Est e Ovest Aulss 8 Berica | UOSD Diabetologia ed Endocrinologia Territoriale | Valdagno (VI) |

| Region | Authors | Hospital | Department | Town |
| --- | --- | --- | --- | --- |
| VENETO | Lucia Gottardo, Valentina Mariano | AULSS 3 Serenissima - Ospedale SS Giovanni e Paolo | UOSD Ipertensione e Patologie Endocrino Metaboliche e Angiologiche | Venezia (VE) |
| VENETO | Anna Maria Letizia Amato, Elisabetta Brun, Silvana Costa, Consuelo Grigoletto, Chiara Alberta Mesturino, Maria Simoncini, Marco Strazzabosco | Ospedale San Bortolo - ULSS 6 Vicenza | UO Endocrinologia e Malattie del Metabolismo | Vicenza (VI) |
| VENETO | Daniela Di Sarra, Beatrice Cenci, Mariarosaria Schioppa, Sara Ciman, Irene Ramponi | CAD Villafranca - Poliambulatori Ospedale Villafranca - AULSS 9 | CAD Villa Franca | Villafranca Veronese (VR) |
| VENETO | Alessandra Marques, Lucia Bondesan | ASL 21 CAD Zevio | Centro Antidiabetico | Zevio (VR) |
