# Supplementary material for: Clinical characteristics and intensification patterns in subjects with “early” type 2 diabetes in Italy- an analysis from the AMD annals initiative
Source: Acta Diabetol. 2025 Oct 30;63(3):453–9. doi: 10.1007/s00592-025-02599-9 (PMC13046648; doi:10.1007/s00592-025-02599-9)
Supplement: Supplementary file 3 — Supplementary Material 3 [file 592_2025_2599_MOESM3_ESM.docx]

**Supplementary Table 1.** Microvascular complications and current glucose-lowering drugs of patients with T2DM in relation to the presence of early diabetes.

| Characteristics | Early diabetes | |
| --- | --- | --- |
|  | **No** | **Yes** |
| Number of patients | 116,756 | 10,700 |
| Albuminuria  Normoalbuminuria  Microalbuminuria  Macroalbuminuria | 67.5  24.7  49.3 | 100  0  0 |
| Glucose-lowering treatment |  |  |
| No pharmachological treatment  Oral agents and/or GLP1-RAs  Insulin  Insulin + Oral agents and/or GLP1-RAs | 1.1  68.8  7.0  28.1 | 13.7  86.3  0.0  0.0 |
| Glucose-lowering drug classes |  |  |
| Metformin | 74.0 | 86.3 |
| Sulphonylureas | 6.2 | 0 |
| Glinides | 0.7 | 0 |
| Pioglitazone | 4.8 | 0 |
| Acarbose | 1.5 | 0 |
| DPPIVi | 21.3 | 0 |
| SGLT2i | 44.6 | 0 |
| GLP1-RAs | 39.5 | 0 |
| Insulin | 35.1 | 0 |
| Basal insulin | 34.4 | 0 |
| Rapid insulin | 15.6 | 0 |
| Eye complications  No retinopathy  Non-proliferative retinopathy  Pre-proliferative retinopathy  Proliferative retinopathy  Blindness  Laser-treated retinopathy  Ophthalmopathy | 85.6  9.3  0.7  0.7  0.2  1.4  2.1 | 100  0  0  0  0  0  0 |
| Foot ulcer/gangrene/osteomyelitis occurring during the period | 0.6 | 0 |
| History of minor lower limb amputation | 0.5 | 0 |
| History of major lower limb amputation | 0.1 | 0 |
| Dialysis | 0.2 | 0 |

Data are expressed as mean±standard deviation or percentages.

**Supplementary table 2**. Add-on to metformin in subjects with early diabetes - trends in BMI and body weight over the years.

| Treatment | Baseline | 12 months | 24 months | 36 months |
| --- | --- | --- | --- | --- |
| Add on of SGLT2i |  |  |  |  |
| BMI (kg/m^2^) | 30.7±5.5 | 30.1±5.4 | 30.1±5.4 | 30.2±5.5 |
| Body weight (kg) | 86.5±17.9 | 84.6±17.6 | 84.9±17.6 | 85.7±18.1 |
| Add on of GLP1-RA |  |  |  |  |
| BMI (kg/m^2^) | 33.3±5.9 | 32.9±5.8 | 32.4±5.6 | 32.7±5.8 |
| Body weight (kg) | 93.8±18.8 | 92.6±18.7 | 92.1±18.7 | 93.1±19.1 |
| Add-on of other glucose-lowering drugs |  |  |  |  |
| BMI (kg/m^2^) | 29.0±5.5 | 28.2±5.1 | 28.7±5.4 | 29.0±5.4 |
| Body weight (kg) | 79.4±16.4 | 77.2±15.9 | 78.6±16.4 | 79.3±16.8 |

Data are expressed as mean±standard deviation

**Supplementary table 3**. Characteristics over time of patients with early T2D receiving metformin monotherapy and not undergoing treatment intensification.

| Characteristics | Baseline | 12 months | 24 months | 36 months |
| --- | --- | --- | --- | --- |
| Number of patients | 22,981 | 15,032 | 11,837 | 9,156 |
| HbA1c (%) | 6.46±0.82 | 6.44±0.75 | 6.56±0.84 | 6.65±0.88 |
| Total cholesterol (mg/dl) | 180.8±39.5 | 174.3±37.6 | 172.3±37.3 | 170.1±36.5 |
| LDL cholesterol (mg/dl) | 105.5±34.4 | 98.2±32.3 | 95.9±32.1 | 94.0±31.5 |
| HDL cholesterol (mg/dl) | 49.6±12.3 | 50.6±12.6 | 50.2±12.6 | 50.2±12.6 |
| Triglycerides (mg/dl) | 133.1±75.1 | 131.9±75.1 | 133.4±72.6 | 133.8±71.4 |
| Systolic blood pressure (mmHg) | 133.3±17.3 | 133.0±17.0 | 133.4±17.0 | 133.8±16.7 |
| Diastolic blood pressure (mmHg) | 78.6±9.6 | 78.3±9.4 | 78.4±9.4 | 78.4±9.5 |
| BMI (kg/m^2^) | 29.6±5.3 | 29.5±5.4 | 29.7±5.4 | 29.7±5.3 |
| Body weight (kg) | 82.2±16.9 | 81.4±16.8 | 81.9±17.0 | 81.9±17.0 |
| eGFR (ml/min) | 86.7±15.9 | 82.3±22.2 | 80.2±23.6 | 78.7±24.2 |
| Subjects with micro/macroalbuminuria | 0 | 12.1 | 14.5 | 15.7 |

Data are expressed as mean±standard deviation or percentages.
